# The Role of Genetics in Stroke Risk and Outcome: A Review of Current Evidence

**DOI:** 10.1002/brb3.70820

**Published:** 2025-10-20

**Authors:** Mega Obukohwo Oyovwi, Benneth Ben‐Azu, Ejayeta Jeroh, Faith B. Friday

**Affiliations:** ^1^ Faculty of Basic Medical Sciences, Department of Human Physiology Delta State University of Science and Technology Ozoro, Delta State Nigeria; ^2^ Faculty of Basic Medical Sciences, Department of Pharmacology DELSU Joint Canada‐Israel Neuroscience and Biopsychiatry Laboratory, Delta State University Abraka, Delta State Nigeria

**Keywords:** cerebrovascular disease, genetics, genome‐wide association studies (GWAS), hemorrhagic stroke, ischemic stroke, personalized medicine, stroke

## Abstract

**Purpose:**

Stroke affects over 15 million people annually, with genetic factors significantly influencing risk and recovery. Understanding the complex interplay of factors contributing to stroke is crucial for developing effective prevention and treatment strategies.

**Methods:**

This review aims to synthesize current evidence regarding the genetic underpinnings of both stroke risk and outcome, encompassing ischemic stroke, hemorrhagic stroke, and specific stroke subtypes. Genetic factors uniquely explain variability in stroke risk and treatment response beyond traditional factors like hypertension. We examine the roles of common genetic variants identified through genome‐wide association studies (GWAS), the influence of rare, high‐impact mutations implicated in monogenic stroke disorders, and the contribution of epigenetic modifications to stroke vulnerability and recovery. Furthermore, we explore the impact of genes involved in key pathways such as coagulation, inflammation, lipid metabolism, and cerebrovascular structure and function.

**Finding:**

This review highlights the growing body of evidence associating specific genetic variants with increased stroke susceptibility, altered stroke severity, and differential responses to treatment. These findings have the potential to refine risk stratification strategies, identify novel therapeutic targets, and personalize stroke management based on individual genetic profiles.

**Conclusion:**

Future research should focus on replicating findings across diverse populations, elucidating gene–environment interactions, and translating genetic discoveries into clinically actionable tools for stroke prevention and improved patient outcomes.

## Graphical abstract

This study delineates the multiple pathways leading to stroke, encompassing genetic risk factors such as lipid/cholesterol metabolism, blood pressure regulation genes, and endothelial dysfunction. The report includes genetic pathways such as Val66Met polymorphism, prothrombin G20210A, interleukin‐6 and tumor necrosis factor gene variant, along with pharmacogenomics pathways that explain drug responses (e.g., aspirin and warfarin), resistance, and prognostic predictors that help assess stroke outcomes. We also included explanations for atherophysiology, oxidative stress, and cell death, as well as epigenetic mechanisms, such as DNA methylation, histone modification, and non‐coding RNAs.

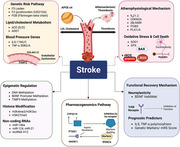

## 1 Introduction

Stroke, a devastating cerebrovascular event, represents a significant global health challenge, demanding ongoing investigation into its intricate etiology and potential therapeutic targets (Bayat et al. 2025; Radenovic and Fischer 2019; Rezaei et al. 2021; Suman et al. 2024; Yadav et al. 2025). Clinically defined as the abrupt interruption of blood supply to the brain, stroke encompasses a spectrum of conditions, broadly categorized into ischemic stroke, hemorrhagic stroke, and transient ischemic attack (TIA) (Myrmel et al. 2025; Zedde et al. 2025). Ischemic stroke, the most prevalent subtype, arises from the occlusion of a cerebral artery, leading to oxygen and nutrient deprivation in the affected brain region (C. Wang et al. 2025). Hemorrhagic stroke, on the other hand, results from the rupture of a blood vessel within the brain parenchyma or surrounding spaces, causing bleeding and subsequent neuronal damage (Iadecola and Anrather 2025). TIA, often referred to as a “mini‐stroke,” involves temporary neurological dysfunction caused by a transient interruption of blood flow, typically resolving within minutes to hours, but serving as a critical warning sign for future, more severe stroke events (Sumathy Bia et al. 2018). Increasingly, genetics is recognized as a critical contributor to stroke susceptibility and outcomes (Rezaei et al. 2021).

The impact of stroke on global health is staggering. It is a leading cause of mortality and long‐term disability worldwide, imposing a substantial burden on healthcare systems and societal resources. Globally, millions of individuals experience stroke annually, with a significant proportion succumbing to its immediate or delayed consequences due to divergent genetic risk factors (Suman et al. 2024). Moreover, stroke survivors often face a multitude of persistent neurological deficits, including motor impairments, speech difficulties, cognitive decline, and emotional disturbances, significantly impacting their quality of life and independence (X. Li, He, et al. 2024). The prevalence of stroke is projected to increase in the coming decades, driven by aging populations and the rising prevalence of modifiable risk factors (Hankey 2020; Torres‐Roman et al. 2025).

Numerous factors contribute to the development of stroke, broadly classified into modifiable and nonmodifiable (Ikenouchi 2025; Johansson et al. 2021). Modifiable risk factors, amenable to lifestyle interventions and medical management, include hypertension, hyperlipidemia, diabetes mellitus, smoking, obesity, physical inactivity, unhealthy diet, and excessive alcohol consumption (Ng et al. 2020). Effective management of these risk factors through public health initiatives and personalized medical care is crucial for primary and secondary stroke prevention (Esenwa and Gutierrez 2015). Nonmodifiable risk factors, such as age, sex, ethnicity, and genetic, are intrinsic to individuals and cannot be directly altered (Mamun et al. 2024). While age is a strong independent predictor of stroke risk, with incidence increasing exponentially with advancing age, sex also plays a role, with men historically exhibiting a higher risk of stroke at younger ages, although this difference appears to narrow in later life (Roy‐O'Reilly and McCullough 2018). Certain ethnic groups also demonstrate a higher predisposition to stroke, highlighting the potential influence of genetic and environmental factors specific to these populations (Ramli et al. 2024).

Beyond these well‐established risk factors, accumulating evidence suggests a significant genetic contribution to stroke risk and outcome (Zeraatiannejad et al. 2023). Genetic factors uniquely explain variability in stroke risk and treatment response beyond traditional factors like hypertension. While stroke is undoubtedly a complex, multifactorial disease influenced by the interplay of environmental exposures and lifestyle choices, the aggregation of stroke within families and the variable response to treatment among individuals point toward an underlying genetic component (Asadabadi et al. 2023). Understanding the genetic architecture of stroke is crucial for identifying individuals at increased risk, predicting disease progression, and ultimately developing targeted therapies that personalize stroke prevention and management strategies. This is particularly important as current treatments are not universally effective and often associated with significant side effects.

This review aims to critically evaluate the current evidence regarding the role of genetics in stroke risk and outcome. By synthesizing findings from genome‐wide association studies (GWAS), candidate gene studies, and other genetic investigations, we will provide a comprehensive overview of the known genetic variants associated with different stroke subtypes, stroke severity, and response to treatment. Furthermore, we will highlight recent advances in the field, including the identification of novel genetic loci and the application of bioinformatics approaches to unravel the complex gene–gene and gene–environment (GxE) interactions that contribute to stroke pathogenesis. Finally, we addressed existing knowledge gaps and proposed directions for future research, emphasizing the need for larger, more diverse genetic studies and translational efforts to translate genetic discoveries into clinically relevant applications. The subsequent sections delved into the genetic basis of ischemic stroke, hemorrhagic stroke, and stroke outcome, followed by a discussion of the challenges and opportunities in translating genetic findings into clinical practice.

## 2 Methods

A systematic literature review was conducted to identify relevant studies investigating the relationship between genetic variants and stroke. The specific methods employed are detailed below:

### 2.1 Search Strategy

A comprehensive search was performed across multiple electronic databases, including PubMed, Embase, and Web of Science, from their inception to 2025. The search strategy incorporated a combination of keywords and controlled vocabulary (MeSH terms where applicable) related to stroke and genetics. These included: “stroke,” “cerebrovascular accident,” “ischemic stroke,” “hemorrhagic stroke,” “intracerebral hemorrhage,” “subarachnoid hemorrhage,” “transient ischemic attack (TIA),” “genetics,” “gene,” “polymorphism,” “single nucleotide polymorphism (SNP),” “mutation,” “variant,” “risk,” “susceptibility,” “outcome,” “prognosis,” “recovery,” “genome‐wide association study (GWAS),” “candidate gene study,” “epigenetics,” “DNA methylation,” and “microRNA.” These terms were combined using Boolean operators (AND, OR) to maximize the sensitivity of the search. An example search string used in PubMed: (“stroke”[MeSH Terms] OR “cerebrovascular accident”) AND (“genetics”[MeSH Terms] OR “gene” OR “polymorphism”) and (“risk” OR “outcome”). The search strategy was adapted for each database to account for differences in indexing and search functionalities. The search was primarily limited to articles published in English. There were no restrictions placed on publication date to ensure a comprehensive capture of relevant literature.

### 2.2 Selection Criteria

*Inclusion Criteria*: Studies were included if they met the following criteria: (1) they were original research articles, meta‐analyses, or systematic reviews; (2) they focused on human subjects; (3) they investigated the association between genetic variants (including single nucleotide polymorphisms (SNPs), copy number variations, and epigenetic modifications) and the risk of stroke (ischemic or hemorrhagic) or stroke outcomes (e.g., mortality, functional disability, recurrence); and (4) they provided sufficient data for analysis.

*Exclusion Criteria*: Studies were excluded if they met any of the following criteria: (1) they were case reports, case series, or editorials; (2) they were animal studies (unless the study provided highly relevant mechanistic insights directly translatable to human stroke); (3) they had small sample sizes (e.g., < 50 participants in case‐control studies or < 100 participants in prospective studies) or exhibited significant methodological flaws compromising the validity of the findings, as determined by quality assessment (see below); and (4) they were reviews without original data or meta‐analyses that did not adequately address the research question.

### 2.3 Data Extraction and Synthesis

Two independent reviewers screened titles and abstracts of identified articles for relevance, based on the predefined inclusion and exclusion criteria. Full‐text articles were obtained for potentially eligible studies and assessed in detail. Discrepancies between reviewers were resolved through discussion and, if necessary, consultation with a third reviewer. Data was extracted from each included study using a standardized data extraction form. Extracted information included: study design (e.g., case‐control, cohort, GWAS, meta‐analysis), sample size, population characteristics (e.g., age, sex, ethnicity), stroke subtype (e.g., large artery atherosclerosis, cardioembolic, small vessel occlusion, hemorrhagic), genetic variants investigated (e.g., specific SNPs, genes, epigenetic markers), genotyping or sequencing methods, outcome measures (e.g., stroke risk, mortality, modified Rankin Scale (mRS) score, Barthel Index), and key findings (e.g., odds ratios [ORs], hazard ratios, *p*‐values).

The evidence was synthesized using a narrative approach, summarizing the key findings from eligible studies. Where appropriate and feasible (e.g., when multiple studies investigated the same genetic variant and stroke outcome), meta‐analysis was considered to quantitatively assess the association. Statistical heterogeneity was assessed using the I2 statistic. The quality of included studies was assessed using appropriate tools, such as the Newcastle‐Ottawa Scale (NOS), for case‐control and cohort studies. Studies were evaluated based on the selection of study groups, comparability of groups, and ascertainment of exposure (case‐control) or outcome (cohort). The NOS was used to assign an overall quality score to each study. The results of the quality assessment were considered when interpreting and synthesizing the evidence.

## 3 Genetics of Stroke Risk

GWAS have emerged as a powerful tool for exploring the genetic landscape of complex diseases like stroke, allowing researchers to identify common genetic variants associated with increased susceptibility (Lee et al. 2025; Yoshimoto et al. 2025; Ikram et al. 2009).

### 3.1 GWAS in Stroke Research

GWAS involves scanning the entire genome of a large number of individuals, typically thousands, to identify SNPs that are more frequently associated with a specific trait or disease (Hettiarachchi and Komar 2022). This approach contrasts with candidate gene studies, which focus on preselected genes based on prior biological knowledge (Ring and Kroetz 2002). In the context of stroke, GWAS compare the genomes of individuals who have suffered from stroke with those who have not, aiming to pinpoint genetic variants that contribute to increased risk (Chauhan et al. 2016). Due to the heterogeneity of stroke, researchers often stratify GWAS by stroke subtype to increase the power to detect subtype‐specific genetic associations (Rosand et al. 2016). Genetic variants that cause ischemic stroke, which is a blockage of the arteries, are PITX2, which predisposes atrial fibrillation (AF), a significant risk factor of cardioembolic stroke (CES) (Franco et al. 2011; Vinciguerra et al. 2024), and the 9p21.3 locus, which enhances atherosclerosis via the dysregulation of CDKN2A/CDKN2B, a predisposing factor of large artery stroke (Ni and Zhang 2014). Conversely, hemorrhagic stroke, caused by rupture of a vessel, is associated with COL4A1 mutations, which cause impairment of the integrity of the vascular basement membrane, and predispose to intracerebral hemorrhage (ICH). This part will tabulate and analyze the main results in various stroke subtypes.

#### 3.1.1 Ischemic Stroke

Ischemic stroke, characterized by blockage of blood flow to the brain, is the most common type of stroke. Several GWAS have successfully identified genetic variants associated with increased risk of ischemic stroke, though the specific genes implicated often differ depending on the population studied and the specific subtype of ischemic stroke being analyzed (Meschia et al. 2011).

9p21.3 Locus: One of the most consistently replicated findings across numerous GWAS is the strong association of the 9p21.3 locus with risk of ischemic stroke, particularly large artery atherosclerotic stroke (LAS) (Meschia et al. 2011; Ni and Zhang 2014). This region contains the CDKN2A/CDKN2B genes, which encode proteins involved in cell cycle regulation and apoptosis (Baker et al. 2016). Initially discovered in coronary artery disease GWAS, the 9p21.3 locus demonstrates a shared genetic susceptibility between cardiovascular and cerebrovascular diseases (Pilbrow et al. 2012). Further fine‐mapping studies have suggested that the noncoding RNA ANRIL (antisense noncoding RNA in the INK4 locus) within this region may play a role in regulating the expression of nearby genes and influencing vascular function (Aznaourova et al. 2020; Fisher et al. 2024).

PITX2 Locus: Another frequently reported association in ischemic stroke GWAS is the PITX2 locus, particularly in CES. The PITX2 gene encodes a transcription factor crucial for cardiogenesis and atrial development (Franco et al. 2011). Genetic variants near PITX2 have been strongly linked to AF, a major risk factor for CES (Vinciguerra et al. 2024). The association between PITX2 and stroke is likely mediated through its influence on AF development (Cruz et al. 2019). Studies have demonstrated that individuals carrying risk alleles in the PITX2 region have an increased risk of developing AF, which in turn increases their risk of CES (Tao et al. 2014).

ZIC4 Locus: Recent GWAS meta‐analyses have identified ZIC4 as a novel locus associated with ischemic stroke risk, particularly in East Asian populations. The ZIC4 gene encodes a transcription factor involved in neural crest development and cerebellar function (Aruga 2004). The specific mechanisms by which ZIC4 influences stroke risk remain to be fully elucidated, but it is hypothesized that it may involve impairments in cerebrovascular development or function (Aruga 2004). Further research is needed to confirm these findings and to investigate the role of ZIC4 in other populations.

#### 3.1.2 Eiden Mutation

**FIGURE 1:**
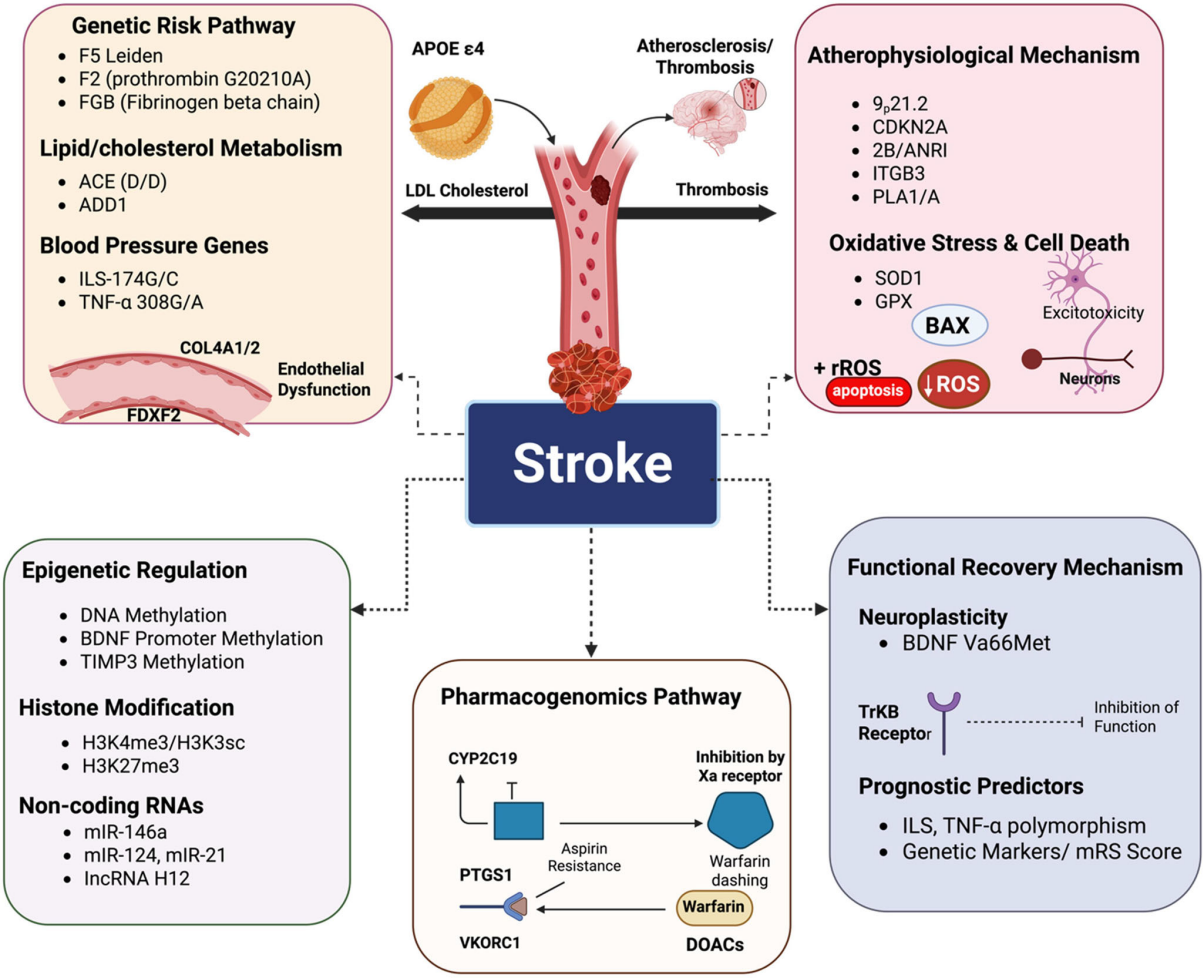
The multiple pathways leading to stroke encompass genetic risk factors such as lipid/cholesterol metabolism, blood pressure regulation genes, and endothelial dysfunction. They also include atherophysiology, oxidative stress, and cell death, as well as epigenetic mechanisms like DNA methylation, histone modification, and noncoding RNAs. Additionally, they cover functional recovery processes, such as neuroplasticity and the BDNF Val66Met polymorphism, along with pharmacogenomics pathways that explain drug responses (e.g., aspirin and warfarin), resistance, and prognostic predictors that help assess stroke outcomes. F2: prothrombin G20210A; FGB: fibrinogen beta chain; ACE D/I: angiotensin‐converting enzyme deletion/insertion; IL 6‐174G/C: interleukin‐6 gene variant; TNF‐α 308G/A: tumor necrosis factor‐alpha gene variant; COL 4A1/2: collagen Type IV alpha 1/2; FDXF 2: ferredoxin 2; SOD1: superoxide dismutase 1; GPX: glutathione peroxidase; BAX: Bcl‐2‐associated X protein; ROS: reactive oxygen species; BDNF Val66Met: brain‐derived neurotrophic factor valine 66 methionine; TrkB: tropomyosin receptor kinase B; CYP2C19: cytochrome P450 2C19; VKORC1: vitamin K epoxide reductase complex subunit 1; DOACs: direct oral anticoagulants; mRS: modified Rankin Scale.

F5 Leiden mutation (rs6025 or F5 p.R506Q) is a point mutation of the Factor V gene, which produces a noncleavable variant of Factor V by the anticoagulant protein activated protein C (APC). The result of this resistance is an extended generation of thrombin and hypercoagulable state and enhances the risk of thrombosis (Figure 1). The mutation is very common in people of European descent, where the carrier frequency is about 5%, but rare in other groups, including African or Asian ancestry. The F5 Leiden mutation, increases thrombotic risk by rendering Factor V resistant to inactivation by APC, leading to a hypercoagulable state. This mutation is strongly associated with venous thromboembolism (VTE) but has a modest link to ischemic stroke, particularly in young adults or those with additional risk factors such as oral contraceptive (OC) use (OR = 1.74, 95% CI: 1.42–2.13). Although F5 Leiden is a well‐recognized risk factor of VTE, comprising deep vein thrombosis (DVT) and pulmonary embolism (PE), its implication in arterial thrombosis, including ischemic stroke, is less obvious (Kujovich 2011). This association has been studied in several studies and meta‐analyses: A 2010 meta‐analysis of 18 case‐control studies of young adults (age 18 years or less; 18 years or less) found that F5 Leiden was associated with ischemic stroke, especially in studies with enrichment of prothrombotic genetic factors (OR = 2.73, 95% CI: 1.98375). The association was weaker and not statistically significant in studies that were not selected (OR = 1.40, 95% CI: 0.9981.95) (Hamedani et al. 2010). In a 2022 meta‐analysis of 104 studies of young adults (18–65 years) Factor V Leiden (FVL) was again a risk factor of ischemic stroke with an OR of 1.74 (95% CI: 1.42213) (Tsalta‐Mladenov et al. 2022).

The Genetics of Early Onset Stroke (GEOS) study, which involved 830 cases of first‐ever ischemic stroke in individuals aged 15–49 years, did not reveal a significant correlation between F5 Leiden and ischemic stroke (OR = 0.94, 95% CI: 0.59–1.50) (Hamedani et al. 2013). These results indicate that the relationship between F5 Leiden and ischemic stroke is small and circumstantial, stronger in younger people or with other risk factors, like the use of OCs or smoking. F5 Leiden affects the choice of anticoagulant treatment clinically because carriers may require dose adjustments or different agents to reduce the thrombotic risk (Hamedani et al. 2013). Nevertheless, screening of stroke patients with F5 Leiden is not advised because this test has a low predictive value for arterial events. It is more relevant in cerebral venous sinus thrombosis cases, where it is more linked with Blood Clotting Factor 5 Leiden. The relationship with F5 Leiden and stroke is not as strong as with VTE, and many carriers never develop stroke. Stroke is ethnically variable and multifactorial, making it challenging in terms of clinical utility. The question of GxE interaction should be further explored in the future to provide a more comprehensive definition of the role.

#### 3.1.3 Angiotensin‐converting enzyme D/D Genotype

The angiotensin‐converting enzyme (ACE) gene insertion/deletion (I/D) polymorphism involves a 287‐base pair fragment in intron 16, with the D allele associated with higher plasma ACE levels. ACE catalyzes the conversion of angiotensin I to angiotensin II, a potent vasoconstrictor, and degrades bradykinin, a vasodilator promoting vasoconstriction and vascular remodeling, which may exacerbate stroke severity (Agerholm‐Larsen et al. 2000; Das et al. 2015). The ACE D/D genotype, linked to hypertension, elevates angiotensin II levels, promoting vasoconstriction and vascular remodeling, and inflammation, all of which are risk factors for stroke (Camós et al. 2012). Meta‐analyses suggest a modest increase in ischemic stroke risk, especially in Asians (OR = 1.37, 95% CI: 1.22–1.53), though findings are inconsistent across populations. The prevalence of the polymorphism varies across populations, with the D allele being more prevalent in Europeans and Asians. ACE D/D genotype has been widely investigated as a risk factor of stroke, with conflicting results: A 1998 meta‐analysis of 7 studies and 1918 Whites showed that the recessive DD genotype is a low‐risk factor of ischemic stroke (OR = 1.31, 95% CI: 1.061.62) (Sharma 1998). A 2012 meta‐analysis of 50 studies covering 10,070 cases of stroke and 22,103 controls, found that DD homozygotes were 37% more likely to develop ischemic stroke than were II and ID genotypes (OR = 1.37, 95% CI: 1.22153), but the effect was stronger in Asians, hospital‐based studies, and small vessel disease (Agerholm‐Larsen et al. 2000; Z. Zhang et al. 2012). An updated meta‐analysis of 105 studies and 47,026 subjects in 2014 confirmed this association, especially in Asians (OR = 1.43, 95% CI: 1.271.61), but only marginally so in Caucasians (OR = 1.14, 95% CI: 1.001.30) (J. Zhao et al. 2014), although no prospect from a 2010 study in Spanish people ACE in Spanish Population (Domingues‐Montanari et al. 2010). Association of the ACE I/D polymorphism with stroke is dependent on population and the effect of this polymorphism is small in comparison to conventional risk factors (Zee et al. 1999). Its effects may be modified by GxE interactions, such as those associated with hypertension or smoking, and this should be studied.

*Brain‐Derived Neurotrophic Factor (BDNF) Val66Met Polymorphism*: BDNF Val66Met variant decreases the production of BDNFs, which worsen the neuroplasticity and the motor recovery after stroke. This polymorphism disrupts the process of activity‐dependent release of BDNF, which influences the synaptic plasticity, as demonstrated in stroke (Cai et al. 2022).

*CYP2C19 Variants*: CYP2C19 loss‐of‐function alleles impair the activation of clopidogrel, diminishing platelet inhibition and causing an elevated risk of the recurrence of stroke. Pharmacogenomic analyses indicate that loss‐of‐function alleles lower the active metabolite of clopidogrel, and this lowers the efficacy of antiplatelet treatment (Saugstad 2015).

*Other Genes and Loci*: Additional genes and loci implicated in ischemic stroke risk through GWAS include ALDH2, associated with alcohol metabolism and potentially contributing to stroke risk through its effects on blood pressure and platelet function (Xu et al. 2019; C. H. Lin et al. 2022; J. Zhang et al. 2023); SHROOM3, linked to vascular smooth muscle cell function and arterial stiffness (W. Liu et al. 2024); and HDAC9, involved in epigenetic regulation and potentially influencing inflammation and vascular remodeling (Das and Natarajan 2020). However, the effect sizes of these associations are generally small, and replication in independent cohorts is often inconsistent.

#### 3.1.4 Hemorrhagic Stroke

Hemorrhagic stroke, resulting from the rupture of a blood vessel in the brain, is less common than ischemic stroke but often carries a higher mortality rate (Runchey and McGee 2010). The genetic architecture of hemorrhagic stroke is distinct from that of ischemic stroke, reflecting the different underlying pathophysiological mechanisms.

*COL4A1/COL4A2 Locus*: Mutations in the COL4A1 and COL4A2 genes, which encode components of Type IV collagen, a major structural protein in basement membranes of cerebral vessels, have been associated with an increased risk of cerebral small vessel disease and hemorrhagic stroke (Guey and Hervé 2022). While these were initially identified through family studies of individuals with early‐onset stroke, subsequent GWAS have provided further evidence for the role of COL4A1 and COL4A2 variants in sporadic hemorrhagic stroke, particularly in individuals with cerebral amyloid angiopathy (CAA) (Debette and Markus 2022; Rannikme et al. 2017).

*FOXF2 Locus*: A GWAS conducted in a Japanese population identified FOXF2, a gene involved in vascular development, as a novel susceptibility gene for ICH (Dofuku et al. 2023). FOXF2 is an important transcription factor for vascular development, and the risk allele was associated with reduced expression levels of the gene, indicating that the genetic variant may alter the vascular integrity of the brain, predisposing individuals to ICH (Ryu et al. 2022).

*EDN1 Locus*: The EDN1 gene, encoding endothelin‐1, a potent vasoconstrictor, has been implicated in the development of vasospasm following subarachnoid hemorrhage (SAH). While not directly associated with the initial rupture, genetic variants near EDN1 have been found to influence the severity of vasospasm and the subsequent risk of ischemic complications after SAH (Griessenauer et al. 2018).

### 3.2 Ethnic Differences in Stroke Genetics

Genetic stroke risk varies across ethnic groups due to differences in allele frequencies and disease mechanisms (Traylor et al. 2012). The ZIC4 locus, identified through GWAS, is more strongly linked to ischemic stroke risk in East Asians (OR = 1.41, 95% CI: 1.28–1.281.56) than in other populations. It may relate to vascular integrity and the regulation of endothelial functions (Traylor et al. 2012). This ethnic‐specific finding underscores the need for diverse genetic studies to address disparities in stroke incidence, which are particularly pronounced in East Asians, especially concerning small vessel disease. Similar associations also vary across populations, with stronger effects seen in Europeans at the PITX2 and 9p21.3 loci concerning CES. These disparities highlight the importance of population‐based research to inform risk assessment and personalized treatment, including targeted screening or tailored therapies, to reduce stroke burden in high‐risk ethnic groups.

## 4 Candidate Gene Studies

Candidate gene studies have been widely conducted to investigate the association between specific genes and stroke risk. These studies typically focus on genes with known biological functions or involvement in relevant pathways such as coagulation, inflammation, and blood pressure regulation (Thakoordeen et al. 2018). By examining the relationship between these genes and stroke, researchers aim to identify potential targets for stroke prevention and treatment.

### 4.1 Genes Involved in Coagulation

Several genes have been implicated in the coagulation pathway and have been studied for their association with stroke risk. These genes include F5 (Factor V), F2 (Prothrombin), and FGB (Fibrinogen Beta Chain) (Stankovic and Majkic‐Singh 2010). The F5 gene has been associated with an increased risk of venous thrombosis and stroke, particularly in the presence of the Leiden mutation (Kujovich 2011). The F2 gene has also been associated with an increased risk of stroke, particularly in the presence of the G20210A mutation (Tatarskyy et al. 2010). The FGB gene, which encodes for the beta chain of fibrinogen, has also been linked to stroke risk, with higher levels of fibrinogen being associated with an increased risk of stroke (Jood et al. 2008).

### 4.2 Genes Involved in Inflammation

Inflammation plays a key role in the pathogenesis of stroke, and several genes involved in the inflammatory response have been studied for their association with stroke risk. These genes include interleukin‐6 (IL‐6) and tumor necrosis factor alpha (TNF‐α) (Nikolic et al. 2020). IL‐6 is a pro‐inflammatory cytokine that has been associated with an increased risk of stroke, particularly in the presence of the ‐174G/C polymorphism (Akinyemi et al. 2017). TNF‐α is another pro‐inflammatory cytokine that has been linked to stroke risk, with higher levels of TNF‐α being associated with an increased risk of stroke (Pawluk et al. 2020).

### 4.3 Genes Involved in Blood Pressure Regulation

Blood pressure regulation is a critical factor in stroke risk, and several genes involved in this pathway have been studied for their association with stroke risk. One such gene is ACE, which plays a key role in the renin‐angiotensin‐aldosterone system (RAAS) (Patel et al. 2017). The ACE gene has been associated with an increased risk of stroke, particularly in the presence of the D/D genotype (Das et al. 2015).

### 4.4 Challenges in Replicating Findings From Candidate Gene Studies

Despite the numerous candidate gene studies conducted to date, there are significant challenges in replicating findings across studies. One major challenge is the issue of rare variants, which may have large effects on stroke risk but are difficult to detect in small sample sizes (Auer and Lettre 2015). Additionally, candidate gene studies often focus on a single variant within a gene, which may not capture the full range of genetic variation within that gene (Rebbeck et al. 2004). Another challenge is the issue of gene–gene interactions, which may contribute to the complex and heterogeneous nature of stroke risk (Feng et al. 2019). Candidate gene studies typically focus on individual genes in isolation, which may overlook the importance of gene–gene interactions in stroke risk.

### 4.5 Rare Variants and Mendelian Stroke Syndromes

In addition to common genetic variants that contribute to stroke risk, there are also rare genetic variants with large effects on stroke risk, often causing monogenic stroke syndromes (Chojdak‐Łukasiewicz et al. 2021). Examples of such syndromes include CADASIL (Cerebral Autosomal Dominant Arteriopathy with Subcortical Infarcts and Leukoencephalopathy) due to NOTCH3 mutations, Fabry disease due to GLA mutations, and CARASIL (Cerebral Autosomal Recessive Arteriopathy with Subcortical Infarcts and Leukoencephalopathy) due to HTRA1 mutations (Ruchoux and Maurage 1997). These rare genetic variants are important to consider in patients with early‐onset stroke or a strong family history (Jaworek et al. 2022; Chalazan et al. 2021). Identifying these variants can help guide clinical management and provide insights into the underlying pathophysiology of stroke.

## 5 GxE Interactions in Stroke

The multifactorial nature of stroke is influenced by both genetic and environmental factors, and their interactions can significantly modulate risk. The concept of GxE interaction posits that the effect of a genetic variant on a phenotype, such as stroke risk, is contingent upon an individual's exposure to specific environmental factors, and vice versa (Nienaber‐Rousseau 2025). This interaction can manifest in several ways. A genetic predisposition may only lead to stroke in the presence of a specific environmental exposure, or the environmental factor may have a more pronounced effect in individuals carrying a particular genetic variant (Boehme et al. 2017). Recognizing these interactions is vital, as it allows for targeted interventions based on an individual's genetic profile and environmental exposures.

### 5.1 Environmental Risk Factors and Their Interaction With Genetic Predisposition

Several well‐established environmental risk factors for stroke, including smoking, hypertension, and diet, are known to interact with genetic factors to influence stroke risk.

*Smoking*: Smoking is a major modifiable risk factor for stroke, contributing to endothelial dysfunction, inflammation, and increased oxidative stress. Studies have shown that the impact of smoking on stroke risk can be influenced by genetic variations in genes involved in nicotine metabolism and cardiovascular function (Larsson et al. 2020). For instance, polymorphisms in the CYP2A6 gene, which encodes a cytochrome P450 enzyme responsible for nicotine metabolism, have been associated with varying levels of nicotine dependence and smoking behavior (Tyndale and Sellers 2002). Individuals carrying certain CYP2A6 variants may be more susceptible to nicotine addiction and, consequently, exhibit a higher risk of stroke due to prolonged and heavier smoking habits (Malaiyandi et al. 2005). Moreover, a study by Morrison et al. (2002) demonstrated that the association between smoking and stroke risk was stronger in individuals carrying a specific variant in the ADD1 gene, which regulates blood pressure. This suggests that genetic predisposition to hypertension, coupled with the pro‐hypertensive effects of smoking, can synergistically elevate stroke risk.

*Hypertension*: Hypertension is a leading risk factor for both ischemic and hemorrhagic stroke. Genetic factors play a significant role in determining an individual's susceptibility to hypertension. Several genes involved in blood pressure regulation, such as those encoding components of the RAAS, have been implicated in hypertension and stroke risk (Seravalle and Grassi 2023). For example, the ACE gene, encoding ACE, has a common insertion/deletion (I/D) polymorphism (Gintoni et al. 2021; Birhan et al. 2022). The DD genotype of the ACE gene has been associated with increased ACE activity and, consequently, higher blood pressure. A meta‐analysis by Zappa et al. (2024) indicated that the association between the ACE I/D polymorphism and stroke risk was stronger in hypertensive individuals compared to normotensive individuals, highlighting a GxE interaction. Further investigation into the interaction between ACE polymorphisms and dietary sodium intake could provide valuable insights, as individuals with certain ACE genotypes might be more sensitive to the blood pressure‐raising effects of sodium.

*Diet*: Dietary factors, including high sodium intake, high‐saturated fat intake, and low intake of fruits and vegetables, are known to increase stroke risk (Micha and Mozaffarian 2010). Genetic variations can influence an individual's response to dietary interventions. For example, polymorphisms in the apolipoprotein E (APOE) gene, which encodes APOE, have been associated with varying levels of cholesterol and triglyceride metabolism (Eichner et al. 2002). Individuals carrying the APOE4 allele tend to have higher cholesterol levels and a greater risk of cardiovascular disease, including stroke (Ward et al. 2009). A study by Sullivan (2020) found that individuals with the APOE4 allele experienced a more pronounced increase in LDL cholesterol levels in response to a high‐fat diet compared to individuals with other APOE genotypes. This suggests that individuals with the APOE4 allele may need to adhere to stricter dietary guidelines to mitigate their increased risk of stroke (Fallaize et al. 2016). Additionally, genetic variations in genes involved in folate metabolism, such as MTHFR, can influence an individual's susceptibility to the detrimental effects of folate deficiency (Hiraoka and Kagawa 2017). The MTHFR C677T polymorphism, a common variant, results in reduced enzyme activity and elevated homocysteine levels, a known risk factor for stroke (L. W. Huang et al. 2022). Studies have shown that the association between the MTHFR C677T polymorphism and stroke risk is stronger in individuals with low folate intake (Chang et al. 2019; L. Zhao et al. 2023).

### 5.2 Specific Examples of GxE Interactions in Stroke

Beyond the broad categories of environmental risk factors, specific examples of GxE interactions in stroke provide a more granular understanding of the complex interplay (Chang et al. 2019; L. Zhao et al. 2023).

*Alcohol Consumption and ALDH2 Polymorphism*: Alcohol consumption is a complex risk factor for stroke, with moderate consumption potentially offering some protection, while heavy consumption increases risk. The ALDH2 gene, encoding aldehyde dehydrogenase 2, plays a critical role in alcohol metabolism (Edenberg 2007). A common variant, ALDH2 *2, results in a nonfunctional enzyme, leading to acetaldehyde accumulation after alcohol consumption, causing flushing and discomfort. East Asian populations have a high prevalence of this variant. Studies have shown that individuals with the ALDH2 *2 allele have a lower risk of heavy alcohol consumption and, consequently, a lower risk of stroke associated with heavy drinking (C. L. Lai et al. 2012). This exemplifies a protective GxE interaction, where a genetic variant influences an individual's behavior (alcohol consumption) and, subsequently, their stroke risk.

*Air Pollution and Genetic Variants in Inflammatory Pathways*: Air pollution is a major environmental risk factor of stroke, and it combines with genetic predispositions to increase the susceptibility of diseases (Kulick et al. 2023). Exposure to air pollution is an increasing environmental risk factor for stroke (Mateen and Brook 2011). Genetic variations in genes involved in inflammatory pathways, such as IL‐6 and TNF‐α, may modify an individual's susceptibility to the adverse effects of air pollution (Dulicek et al. 2018). Air pollution, particularly fine particulate matter (PM2.5), triggers oxidative stress and systemic inflammation, which worsen endothelial dysfunction and atherosclerosis in individuals with genetic risk factors for inflammatory processes, including IL‐6 rs1799963 (Roy‐O'Reilly and McCullough 2018). For instance, other studies have shown that the risk of stroke in carriers of IL‐6 variants exposed to high levels of PM2.5 is significantly higher (OR = 1.45, 95% CI: 1.32160) compared to noncarriers in low‐exposure areas (Panasevich et al. 2013). A study by (Panasevich et al. 2013) found that individuals carrying specific variants in the IL‐6 gene had a greater increase in blood pressure and inflammatory markers in response to air pollution exposure compared to individuals with other IL‐6 genotypes (Dulicek et al. 2018). This suggests that genetic predisposition to inflammation can exacerbate the detrimental effects of air pollution on cardiovascular health and increase stroke risk. Similarly, oxidative stress gene variants can elevate the risk of vascular damage caused by PM2.5, including certain NQO1 variants (Zeraatiannejad et al. 2023). Such interactions highlight the importance of combining genetic and environmental data in stroke prevention, encouraging measures like air quality regulation and genetic risk assessment to protect vulnerable populations.

*OC Use and FVL*: OC use is a known risk factor for VTE, which, in rare cases, can lead to stroke (Dulicek et al. 2018). FVL is a common genetic mutation that increases the risk of VTE. The combination (Miller et al. 2017) of OC use and FVL significantly elevates the risk of VTE and, consequently, stroke (Reddy et al. 2022; Dayan et al. 2011). This represents a synergistic interaction, where two independent risk factors combine to produce a much greater risk than the sum of their individual effects.

## 6 Epigenetics and Stroke Risk

Epigenetics refers to heritable changes in gene expression that occur without alterations to the underlying DNA sequence (Ajayi et al. 2024). These modifications, including DNA methylation, histone modification, and noncoding RNA regulation, play a crucial role in regulating gene expression patterns in response to environmental stimuli and developmental cues. Accumulating evidence suggests that epigenetic modifications are involved in the pathogenesis of stroke and may contribute to individual differences in stroke susceptibility and outcome (Stanzione et al. 2020).

### 6.1 Epigenetic Modifications

*DNA Methylation*: DNA methylation is the addition of a methyl group to a cytosine base, typically at CpG dinucleotides. DNA methylation is generally associated with gene silencing, although its effect can vary depending on the genomic context. Changes in DNA methylation patterns have been observed in various diseases, including stroke. Studies have shown that stroke can induce alterations in DNA methylation patterns in brain tissue, blood cells, and other tissues (Choi et al. 2022). For example, a study found that DNA methylation levels at specific CpG sites in the BDNF gene, which encodes BDNF, were altered in stroke patients compared to healthy controls (Xie et al. 2017). BDNF is a crucial neurotrophic factor involved in neuronal survival and plasticity. These methylation changes were associated with altered BDNF expression and poorer functional outcomes after stroke. Furthermore, environmental factors, such as smoking and diet, can influence DNA methylation patterns and, consequently, stroke risk. A prospective cohort study by (Richmond et al. 2015) found that maternal smoking during pregnancy was associated with altered DNA methylation patterns in offspring, potentially increasing their risk of cardiovascular diseases, including stroke, later in life (Rogers 2019).

*Histone Modification*: Histone modifications are also important in the pathophysiology of stroke beyond DNA methylation. Histone modifications involve the addition of chemical groups, such as acetyl groups or methyl groups, to histone proteins, which package DNA into chromatin (Ajayi et al. 2024). These modifications can alter chromatin structure and accessibility, thereby influencing gene transcription. Histone acetylation is generally associated with increased gene expression, while histone methylation can have either activating or repressing effects depending on the specific histone residue modified (Rahman et al. 2004). Histone acetylation, catalyzed by histone acetyltransferases (HATs) and histone deacetylases (HDACs), usually activates genes, while histone methylation, catalyzed by histone methyltransferases (HMTs), can either activate or repress genes depending on the residue involved and the methylation state (Morris‐Blanco et al. 2022). Studies have shown that stroke can induce changes in histone modification patterns in brain tissue, influencing gene expression and neuronal survival (Su et al. 2022). In stroke, such changes regulate major processes including inflammation, apoptosis, and neuroprotection. For example, histone H3K4 trimethylation (H3K4me3) of the IL‐6 promoter causes an increase in IL‐6 levels, promoting poststroke inflammation, while histone H3K27 trimethylation (H3K27me3) of IL‐6 may silence its expression and reduce inflammatory damage (Zeraatiannejad et al. 2023; Lee et al. 2025). Similarly, histone H3K9 acetylation (H3K9ac) at the BDNF promoter boosts BDNF expression, aiding neuroprotection and recovery (Ikram et al. 2009). Apoptosis is also influenced by histone modifications, where H3K27me3 at the BCL‐2 promoter suppresses anti‐apoptotic signaling, worsening neuron death. HDAC inhibitors have shown potential as therapies in preclinical stroke models, since valproic acid and vorinostat have been demonstrated to decrease inflammation and enhance neuroprotection by promoting acetylation of neuroprotective gene promoters (Fessler et al. 2013). A study by found that HDAC inhibitors, which promote histone acetylation, improved functional outcomes in experimental stroke models (Tang et al. 2017). This suggests that alterations in histone acetylation play a role in stroke pathology and that targeting histone modifications may offer therapeutic benefits. Also, inhibitors of HMTs, including EZH2, which catalyzes H3K27me3, might stimulate the expression of neuroprotective genes, which provides new opportunities of treatment (Luo et al. 2020). Similar to DNA methylation, histone modification patterns can be influenced by environmental factors. For instance, dietary interventions, such as caloric restriction, have been shown to alter histone modification patterns and improve neuronal function in experimental models (Pani 2015). These results indicate that histone changes can be used to treat stroke.

#### 6.1.1 Noncoding RNAs

Noncoding RNAs exist in the form of microRNAs (miRNAs) and long noncoding RNAs (lncRNAs) and are important in the pathophysiology of stroke (Cai et al. 2022). As an example, miR‐146a, which acts on the inflammation through the NF‐kB pathway, is downregulated in the patients with ischemic stroke, which can contribute to the increase of inflammatory damage (Bao et al. 2018). Likewise, lncRNA H19 is overexpressed in patients with stroke (Mahjoubin‐Tehran et al. 2021), and it is related to a particular subtype of stroke, indicating its role in stroke pathogenesis. Additional ncRNAs, including miR‐124 and miR‐21, have been observed to play roles in neuroprotection and apoptosis following stroke (Saugstad 2015), indicating that ncRNAs have a wide range of effects on stroke outcome. ncRNAs is important although they affect gene expression on the RNA level, but do not encode proteins, and therefore affect many processes, such as inflammation, apoptosis, and neuroprotection, that are key to understanding stroke pathophysiology. An example is MiR‐146a that has been reported to inhibit tumor necrosis factor receptor‐associated factor 6 (TRAF6) and interleukin‐1 receptor‐associated kinase 1 (IRAK1), which are major constituents of the NF‐kB pathway, which becomes activated during stroke‐induced inflammation (Cai et al. 2022). Its inhibition, as it is the case in patients with ischemic stroke, could lead to increasing inflammatory processes, which could further exacerbate results.

Conversely, lncRNA H19 has been demonstrated to foster neuroinflammation and inhibit neurogenesis, exposing individuals to atherosclerosis when exposed to hypoxic conditions (Mahjoubin‐Tehran et al. 2021). Since it is upregulated and linked to certain subtypes of strokes (e.g., SVO, LAA), it is possible that it could be a biomarker of diagnosis, with receiver operating characteristic (ROC) analysis showing that it is highly sensitive and specific in the first 24 h after the stroke (Bao et al. 2018). MiR‐124 is neuroprotective, increases neuronal survival, and decreases apoptosis, whereas miR‐21 plays a role in angiogenesis and neuroprotection, which are essentials in poststroke recovery.

### 6.2 DNA Methylation in Stroke

Several studies have investigated DNA methylation changes associated with stroke, focusing on different tissues and clinical outcomes (Jiménez‐Balado et al. 2024; Cullell et al. 2022).

*Blood‐Based Epigenetic Markers*: Peripheral blood samples offer a convenient and noninvasive source of DNA for epigenetic analysis. Studies have identified several blood‐based DNA methylation markers associated with stroke risk and outcome. For example, a study by Agha et al. (2019) identified a DNA methylation signature in blood associated with incident stroke in a large population‐based cohort. This signature involved methylation changes in genes related to inflammation, lipid metabolism, and vascular function. These findings suggest that blood‐based DNA methylation markers can be used to identify individuals at increased risk of stroke and to monitor the effects of preventive interventions. However, it is crucial to acknowledge that DNA methylation patterns in blood may not perfectly reflect those in brain tissue, the primary site of stroke injury.

*Brain Tissue Epigenetic Analysis*: Analyzing DNA methylation patterns in brain tissue provides a more direct assessment of epigenetic changes associated with stroke pathology. However, obtaining brain tissue samples is often limited to postmortem studies or biopsy samples in specific clinical scenarios. Studies analyzing brain tissue from stroke patients have identified DNA methylation changes in genes involved in neuronal survival, inflammation, and angiogenesis (Phillips et al. 2023). For instance, a study by Shcherbak et al. (2022) found that DNA methylation levels at specific CpG sites in the TIMP3 gene, encoding tissue inhibitor of metalloproteinases 3, were altered in peri‐infarct brain tissue stroke. TIMP3 plays a role in regulating extracellular matrix remodeling and angiogenesis (Basu et al. 2013). These methylation changes were associated with altered TIMP3 expression and poorer functional outcomes.

*Epigenome‐Wide Association Studies (EWAS)*: EWAS are hypothesis‐free approaches that examine DNA methylation patterns across the entire genome to identify regions associated with a specific phenotype, such as stroke risk or outcome. Several EWAS have been conducted in stroke, identifying novel DNA methylation markers associated with the disease (Jiménez‐Balado et al. 2024). For example, a meta‐analysis of EWAS studies by Carbonneau et al. (2024) identified several replicated DNA methylation sites associated with stroke risk, highlighting the importance of large‐scale collaborative efforts in epigenetic research.

## 7 Pharmacogenomics

Stroke is a complex condition often resulting from thromboembolic events. Antiplatelet and anticoagulant medications play a crucial role in both the primary and secondary prevention of stroke by inhibiting clot formation (Siasos et al. 2020). However, the effectiveness of these medications can vary considerably among individuals. This variability has spurred significant interest in pharmacogenomics, the study of how genes affect a person's response to drugs. Understanding the genetic factors that influence drug metabolism, drug targets, and other pharmacological pathways can lead to personalized stroke prevention strategies, optimizing treatment efficacy and minimizing adverse events (Georgakis and Mendelian 2021).

### 7.1 The Promise of Pharmacogenomics in Stroke Prevention

The core principle underpinning pharmacogenomics is that genetic variations, also known as polymorphisms, can alter the expression, structure, or function of proteins involved in drug disposition and action (Qahwaji et al. 2024). These variations can affect a drug's absorption, distribution, metabolism, and excretion (ADME), leading to altered plasma concentrations and, consequently, varying degrees of therapeutic response (Tibbitts et al. 2016). Furthermore, genetic variations in the drug target itself, or in proteins involved in downstream signaling pathways, can influence the effectiveness of a drug at its intended site of action (Landry and Gies 2008).

In the context of stroke prevention, the application of pharmacogenomics aims to identify individuals who may be less responsive to standard antiplatelet or anticoagulant therapies, allowing for tailored treatment regimens. This personalized approach holds the promise of improving clinical outcomes, reducing the risk of stroke recurrence or initial occurrence, and minimizing the potential for bleeding complications (Spertus et al. 2015).

### 7.2 Clopidogrel and CYP2C19: A Case Study in Pharmacogenomics

Clopidogrel, a thienopyridine antiplatelet agent, is widely used to prevent thrombotic events in patients with acute coronary syndrome, peripheral artery disease, and stroke (Patti et al. 2020). However, clopidogrel is a prodrug, meaning it requires metabolic activation in the liver to exert its antiplatelet effects (Sangkuhl et al. 2010). This activation is primarily mediated by the cytochrome P450 (CYP) enzyme system, particularly CYP2C19 (Manikandan and Nagini 2017). The *CYP2C19* gene exhibits significant genetic polymorphism, with numerous variant alleles identified across different populations. These alleles are typically classified based on their impact on enzyme activity, ranging from *loss‐of‐function* alleles, which result in reduced or absent enzyme activity, to *gain‐of‐function* alleles, which enhance enzyme activity (Vihinen 2021). Individuals carrying *loss‐of‐function* alleles, termed “poor metabolizers” (PMs), exhibit reduced activation of clopidogrel, leading to lower levels of the active metabolite and diminished antiplatelet effects (O'Connor et al. 2012). Conversely, individuals carrying *gain‐of‐function* alleles, termed “ultrarapid metabolizers” (UMs), tend to have increased activation of clopidogrel and potentially an increased risk of bleeding (Hassani Idrissi et al. 2018). Intermediate metabolizers (IMs) and normal metabolizers (NMs) possess varying levels of CYP2C19 activity based on their specific allele combinations.

Numerous studies have demonstrated a clear association between *CYP2C19* genotype and clopidogrel response. The landmark TRITON‐TIMI 38 trial, for example, showed that carriers of *CYP2C19* loss‐of‐function alleles experienced a higher rate of cardiovascular death, myocardial infarction (MI), or stroke compared to noncarriers, particularly in patients undergoing percutaneous coronary intervention (PCI) (Mega et al. 2010). A meta‐analysis of multiple studies further confirmed that *CYP2C19* PMs had a higher risk of stent thrombosis and major adverse cardiovascular events (MACE) when treated with standard‐dose clopidogrel (Mega et al. 2010). The impact of *CYP2C19* polymorphisms on clopidogrel response in stroke patients has also been investigated. Studies have shown that *CYP2C19* loss‐of‐function alleles are associated with a higher risk of recurrent stroke or other vascular events in patients treated with clopidogrel after an initial stroke or TIA (McDermott et al. 2022; Cargnin et al. 2024). A systematic review and meta‐analysis by Cargnin et al. (2024) found that *CYP2C19* loss‐of‐function alleles were significantly associated with an increased risk of stroke recurrence in East Asian populations receiving clopidogrel, highlighting the importance of considering ethnic variations in genetic testing.

### 7.3 Clinical Implications and Alternative Strategies

The clinical implications of *CYP2C19* pharmacogenomics are significant. Identifying *CYP2C19* PMs, IMs, and UMs can allow clinicians to tailor antiplatelet therapy to individual patients, potentially improving efficacy and safety (Gower et al. 2020). Several strategies have been proposed to mitigate the impact of *CYP2C19* polymorphisms on clopidogrel response:

*Increased Clopidogrel Dose*: One approach is to increase the dose of clopidogrel in *CYP2C19* PMs to compensate for the reduced activation of the drug. However, studies evaluating this strategy have yielded mixed results, with some showing improved antiplatelet effects but also an increased risk of bleeding (Serebruany et al. 2004).

*Alternative Antiplatelet Agents*: A more commonly accepted strategy is to switch to an alternative antiplatelet agent that is less dependent on CYP2C19 for activation. Prasugrel and ticagrelor are two examples of newer antiplatelet agents that have shown greater efficacy than clopidogrel, particularly in patients with *CYP2C19* loss‐of‐function alleles (Claassens et al. 2021). However, these agents are generally associated with a higher risk of bleeding compared to clopidogrel.

*Genotype‐Guided Therapy*: Genotype‐guided therapy, where the choice of antiplatelet agent is based on an individual's *CYP2C19* genotype, is gaining increasing acceptance (Gower et al. 2020). Several studies have demonstrated the feasibility and potential benefits of this approach. For example, the POPular Genetics trial showed that genotype‐guided antiplatelet therapy was associated with a reduction in bleeding events compared to standard therapy in patients undergoing PCI (Claassens et al. 2021).

### 7.4 Clinical Applications of Genetic Testing in Stroke Care

The study of stroke genetics is very promising in the context of reducing the suffering of patients and improving health care systems worldwide. Detection of genetic risk factors enables clinicians to apply early intervention measures to avoid a stroke in high‐risk patients, including lifestyle changes or specific treatments. As an example, the personalized antiplatelet treatment can be informed with genetic testing of variants such as CYP2C19, alleviating recurrent stroke by up to 27% in patients with certain genetic backgrounds (Y. Wang et al. 2021). Such precision medicine is not only useful in enhancing patient outcomes but also in maximizing healthcare resource distribution so that more effective utilization of healthcare services is achieved. Moreover, genetic knowledge can lead to the innovation in stroke management and the creation of new diagnostics and therapeutics that could be adopted in the healthcare systems of many countries. The multidisciplinary cooperation must remain consistent in the translation of these genetic discoveries into just, affordable interventions that can be of benefit to various population groups (Table 1).

**TABLE 1:** Pharmacogenomic variants and clinical implications of antithrombotic drugs in stroke management.

Drug	Associated gene	Polymorphisms	Clinical implications
Clopidogrel	CYP2C19	2, 3 (loss‐of‐function), 17 (gain‐of‐function)	Loss‐of‐function alleles (Suman et al. 2024; Bayat et al. 2025) reduce clopidogrel activation, increasing recurrent stroke risk (RR = 1.92, 95% CI: 1.57–2.35 for IM/PMs). Genotype‐guided therapy with alternatives like ticagrelor reduces recurrence (HR = 0.77, 95% CI: 0.64–0.94) (Y. Wang et al. 2016; Pan et al. 2017).
Aspirin	PTGS1, GPVI, ITGB3	Various SNPs	Polymorphisms may lead to aspirin resistance, reducing effectiveness in stroke prevention (Goodman et al. 2008; C. X. Li, Sun, et al. 2024). No specific genotype‐guided strategies are established.
Warfarin	VKORC1, CYP2C9	VKORC1 haplotypes, CYP2C9 variants	Variations affect dose requirements, with genotype‐guided dosing improving anticoagulation control and reducing bleeding risk (Kimmel et al. 2013; Dean 2018).
DOACs (e.g., dabigatran, rivaroxaban, apixaban, edoxaban)	ABCB1, CYP3A4, CYP3A5	Variants in ABCB1, CYP3A4, CYP3A5	Variations may influence plasma concentrations, but clinical significance is unclear, requiring further research (Gong and Kim 2013; Huppertz et al. 2019).

Genotyping CYP2C19 is potentially very useful in personalizing antiplatelet treatment in stroke patients and especially those receiving clopidogrel as a secondary preventative measure. The low metabolizers (PMs) and IMs with loss‐of‐function alleles (2 or 3) have lower clopidogrel activation and a nearly twofold higher risk of recurrent stroke (relative risk [RR] = 1.92, 95% CI: 1.5712.35, *p* < 0.001) than extensive metabolizers (EMs) (Maas et al. 2024). They can screen these high‐risk patients by genotyping and switch them to other P2Y12 inhibitors such as ticagrelor that have been shown to reduce the risk of stroke recurrence in CYP2C19 LoF carriers (hazard ratio [HR] = 0.77, 95% CI: 0.640.94, *p* = 0.008) (Y. Wang et al. 2021). In addition to the application of pharmacogenomics, genetic risk variant testing (e.g., 9p21.3), may be used to support risk stratification, and drive preventive interventions (e.g., intensified lipid‐lowering therapy) (Anderson et al. 2010). Nevertheless, these issues like high cost of genetic testing, low accessibility in low resource environments, and standardized protocols in low‐income countries prevent its widespread use (Owolabi et al. 2015). The inclusion of genetic testing in clinical practice may be the best method of managing stroke, although these barriers are central to equal implementation.

### 7.5 Pharmacogenomics of Other Antiplatelet and Anticoagulant Agents

While the relationship between *CYP2C19* and clopidogrel is the most well‐studied example of pharmacogenomics in stroke prevention, other genetic variations can also influence the response to antiplatelet and anticoagulant medications.

#### 7.5.1 Aspirin

Aspirin's antiplatelet effect is mediated by the irreversible inhibition of cyclooxygenase‐1 (COX‐1), which is encoded by the *PTGS1* gene. Polymorphisms in *PTGS1* have been associated with varying degrees of aspirin responsiveness (Dawidowicz et al. 2020). Furthermore, variations in genes involved in platelet aggregation, such as *GPVI* and *ITGB3*, have also been linked to aspirin resistance (da Silva et al. 2023) (Figure 1).

#### 7.5.2 Warfarin

Warfarin is a vitamin K antagonist used for anticoagulation in patients with AF and other conditions predisposing to stroke. Warfarin's mechanism of action involves inhibiting the vitamin K epoxide reductase complex subunit 1 (VKORC1), which is encoded by the *VKORC1* gene. Genetic variations in *VKORC1* are a major determinant of warfarin dose requirements, with certain haplotypes associated with lower or higher dose requirements (Obayashi et al. 2006). Polymorphisms in *CYP2C9*, which metabolizes warfarin, also contribute to interindividual variability in warfarin response (Takahashi and Echizen 2003). Genotype‐guided warfarin dosing algorithms are now widely available and have been shown to improve anticoagulation control and reduce the risk of bleeding (Tse et al. 2018).

*Direct Oral Anticoagulants (DOACs)*: DOACs, such as dabigatran, rivaroxaban, apixaban, and edoxaban, are increasingly used as alternatives to warfarin for stroke prevention in AF. While pharmacogenomic studies of DOACs are still in their early stages, some evidence suggests that genetic variations in genes encoding drug transporters, such as *ABCB1*, and metabolizing enzymes, such as *CYP3A4* and *CYP3A5*, may influence DOAC plasma concentrations and clinical outcomes (Shnayder et al. 2021). However, the clinical significance of these findings remains to be fully elucidated (Table 1).

## 8 Genetics of Stroke Outcome

While the acute management of stroke has improved significantly, predicting individual outcomes remains a significant challenge. The severity of the initial stroke insult and the subsequent functional recovery are crucial determinants of long‐term disability and quality of life. Understanding the genetic architecture underlying these outcomes is crucial for developing personalized strategies for stroke prevention, diagnosis, and rehabilitation.

### 8.1 Genetic Predictors of Stroke Severity

Stroke severity, often quantified using the National Institutes of Health Stroke Scale (NIHSS), reflects the extent and location of brain damage sustained during the acute event. The NIHSS is a standardized neurological examination tool assessing various neurological functions, including consciousness, language, motor function, and sensory perception (Iacono et al. 2014). Higher NIHSS scores indicate more severe deficits and are generally associated with poorer outcomes (Mihindu et al. 2019). Identifying genetic variants associated with stroke severity could potentially enable early risk stratification, guiding more aggressive interventions in individuals predisposed to severe stroke outcomes. Several studies have investigated genetic associations with NIHSS scores, focusing on candidate genes involved in various pathways relevant to stroke pathophysiology, including inflammation, thrombosis, excitotoxicity, and neuroprotection (Qin et al. 2022; Maida et al. 2024).

#### 8.1.1 Inflammation‐Related Genes

Inflammation plays a critical role in the pathophysiology of stroke, contributing to secondary brain injury and exacerbating the initial ischemic damage (Amantea et al. 2009). Genetic variations in genes encoding inflammatory mediators and receptors have been explored as potential predictors of stroke severity.

*Interleukin‐6 (IL‐6)*: IL‐6, a pro‐inflammatory cytokine, is significantly upregulated after stroke and contributes to neuronal damage. A study by Cui et al. (2012) and H. Zhao et al. (2017) investigated the association between IL‐6 promoter polymorphisms and stroke severity in a Chinese population. They found that the ‐174G/C polymorphism was significantly associated with NIHSS score, with the G allele associated with more severe strokes. This finding suggests that individuals carrying the G allele may mount a more pronounced inflammatory response, leading to increased brain damage.

*TNF‐α*: It is another key pro‐inflammatory cytokine implicated in stroke pathogenesis. Variations in the TNF‐α promoter region, particularly the ‐308G/A polymorphism, have been investigated in relation to stroke severity. A meta‐analysis by Wu et al. (2019) found a significant association between the TNF‐α‐308A allele and increased risk of severe stroke, suggesting that this allele may contribute to a more robust inflammatory response and greater brain damage. Conversely, other studies have reported no significant association (L. Li et al. 2014) potentially due to differences in study design, population characteristics, and sample size.

*Interleukin‐1 beta (IL‐1β)*: The association between IL‐1β+3954 C/T polymorphism and stroke severity is inconclusive due to conflicting study results, potentially stemming from variations in study populations, stroke subtypes, and genetic backgrounds. IL‐1β plays a role in stroke pathophysiology by driving neuroinflammation and exacerbating ischemic damage, with elevated levels correlating with infarct volume (Sobowale et al. 2016; Murray et al. 2015). Studies in sickle cell disease (SCD) have linked the IL‐1β+3954 TT genotype to pulmonary hypertension (Afifi et al. 2019), while IL‐1β promoter polymorphisms are associated with increased ICH risk in brain arteriovenous malformation patients (Murray et al. 2015). However, a meta‐analysis of CHD cases found no association between the IL‐1β+3954 polymorphism and cardiovascular risk (L. Zhou et al. 2012), and previous stroke meta‐analyses have also failed to demonstrate significant associations with IL‐1 cluster polymorphisms (L. Zhou et al. 2012). Population stratification, stroke subtype, sample size, and GxE interactions can influence results, with strong associations in SCD (Afifi et al. 2019) contrasting with null results in general stroke (L. Zhou et al. 2012), and differential effects observed in ischemic versus hemorrhagic stroke (Murray et al. 2015). Although IL‐1β contributes to stroke pathophysiology (Sobowale et al. 2016; Murray et al. 2015), current evidence does not strongly support IL‐1β+3954 C/T as a universal predictor of stroke severity, but it may modify risk in specific subpopulations (Afifi et al. 2019). Validation in large, phenotypically stratified cohorts is needed.

#### 8.1.2 Thrombosis‐Related Genes

Thrombosis plays a crucial role in the etiology of ischemic stroke, and genetic variations affecting coagulation and platelet function could influence stroke severity by affecting the extent and duration of cerebral ischemia. Though uncommon, understanding the pathogenetic mechanisms of rare stroke syndromes can significantly increase the refinement of the diagnostic approach and development of novel therapies improved for stroke management.

*FVL*: The FVL, a genetic variant causing APC resistance, shows a complex relationship with arterial thrombosis and ischemic stroke risk. While strongly linked to VTE, its arterial implications remain debated. Meta‐analyses reveal FVL doubles ischemic stroke risk in young adults (OR 2.00; 95% CI 1.59–2.51), but this association is stronger in studies selecting patients with suspected prothrombotic states (OR 2.73) compared to unselected populations (OR 1.40) (Hamedani et al. 2010). Large cohort studies, including the GEOS study of 15–49‐year‐olds, found no significant association in general adult populations (3.6% FVL frequency in stroke patients vs. 3.8% in controls) (Hamedani et al. 2013). When combined with other inherited thrombophilias (e.g., protein C/S deficiency), FVL increases arterial ischemic stroke risk (pooled OR 1.25) (Chiasakul et al. 2019). In 69,681 patients with established coronary heart disease, FVL showed no association with recurrent MI, stroke, or mortality (HR 1.03 for primary outcomes) (Mahmoodi et al. 2020). Case reports highlight FVL's potential role in arterial thrombosis when combined with hyperhomocysteinemia or peripheral arterial disease (Page et al. 2005). Homozygous FVL in mice accelerates arterial thrombosis (27 vs. 56 min to occlusion in wild‐type) and exacerbates atherosclerosis (Eitzman et al. 2005), though human relevance remains uncertain. The provided studies do not directly address stroke severity or infarct volume. While earlier research cited in the query suggests conflicting results, current meta‐analyses and cohort studies (Hamedani et al. 2013; Chiasakul et al. 2019; Mahmoodi et al. 2020) focus on incidence rather than outcomes, leaving this question unresolved in the available literature. FVL testing may be considered in young stroke patients with suspected prothrombotic states (1), but routine screening is not supported for arterial risk stratification in adults with established cardiovascular disease (Mahmoodi et al. 2020). Management should prioritize controlling traditional risk factors (e.g., hypertension, hyperlipidemia) over thrombophilia testing in most arterial thrombosis cases (190,192).

*Prothrombin G20210A*: The prothrombin G20210A mutation, a genetic variant mainly linked to increased VTE risk, has a controversial role in arterial events like ischemic stroke, with conflicting study results. (1) Studies suggest an association between the mutation and ischemic stroke in young adults (15–49 years), particularly those aged 15–42 years, with meta‐analyses indicating increased stroke risk in adults ≤ 55 years (OR = 1.4–1.5), but these findings require replication (Jiang et al. 2014). (2) Research in Iran and Iraq found no significant association between the mutation and ischemic stroke in their populations, suggesting it may not independently increase stroke risk without other factors like hypertension or diabetes (Saadatnia et al. 2012; S. A. Ahmed et al. 2020). (3) Stroke risk modifiers, such as smoking, OC use, and migraine, may amplify the mutation's impact on stroke risk in certain subgroups (Jiang et al. 2014). While the mutation is a well‐established risk factor for venous thrombosis, its role in arterial thrombosis, including stroke and MI, is less clear and appears weaker (Saadatnia et al. 2012; C. Li et al. 2017).

*Platelet Glycoprotein IIIa (GPIIIa) PLA1/A2 Polymorphism*: The Platelet Glycoprotein IIIa (GPIIIa) PlA1/A2 polymorphism, a genetic variation involving a leucine‐proline substitution at position 33 of the GPIIIa subunit, has been extensively studied for its potential role in cardiovascular diseases, including stroke (Radenovic and Fischer 2019; Suman et al. 2024). GPIIIa is part of the GPIIb‐IIIa complex, the most abundant integrin on the platelet surface, which plays a crucial role in platelet aggregation by binding fibrinogen, von Willebrand factor, and fibronectin to help terminate bleeding after vascular injury (Floyd et al. 2014; M. A. M. Ahmed et al. 2024). The PlA1/A2 polymorphism has been associated with altered platelet function and potentially increased cardiovascular risk (Floyd et al. 2014; M. A. M. Ahmed et al. 2024). Some studies suggest the PlA2 allele may be associated with an increased risk of ischemic stroke, possibly due to enhanced platelet aggregation (Coadă et al. 2024), though findings are inconsistent. The impact of the PlA1/A2 polymorphism on stroke severity remains unclear, with some studies reporting no significant effect and others suggesting potential associations with increased risk (Coadă et al. 2024). The PlA2 allele has also been linked to an increased risk of MI, with meta‐analyses showing a significant association (Floyd et al. 2014; M. A. M. Ahmed et al. 2024). Despite initial suggestions, recent meta‐analyses indicate that the PlA2 allele is not a reliable biomarker for resistance to antiplatelet drugs (Floyd and Ferro 2014).

#### 8.1.3 Excitotoxicity and Neuroprotection‐Related Genes

Excitotoxicity, primarily mediated by glutamate, contributes to neuronal damage following stroke. Genes involved in glutamate metabolism, neurotrophic factors, and antioxidant defense mechanisms have been investigated as potential predictors of stroke severity.

*Glutamate Receptor Genes*: Glutamate receptors, particularly NMDA receptors, are central to excitatory neurotransmission and are implicated in excitotoxic neuronal damage, a key factor in stroke pathophysiology (Nicolo et al. 2019). During ischemic stroke, glutamate levels rise, contributing to neuronal death via NMDA receptor overactivation (K. Lai et al. 2024). Variations in genes encoding these receptors have been explored for their potential influence on stroke severity, but this research is limited and inconclusive. Glutamate signaling involves multiple receptor types and complex interactions, complicating the isolation of specific genetic variation effects (Hakon et al. 2024). NMDA receptor antagonists have failed in clinical trials due to side effects, highlighting the need for targeted approaches (K. Lai et al. 2024; Hansen et al. 2017). Future research into more specific targets, like acid‐sensing ion channels (ASICs) modulated by glutamate, may offer new stroke treatments without the side effects of NMDA receptor blockade (K. Lai et al. 2024). Inhibiting metabotropic glutamate receptors like mGluR5 has shown potential in restoring sensorimotor functions poststroke, suggesting another therapeutic pathway (Hakon et al. 2024). Overall, while glutamate receptors are critical in stroke pathophysiology, further research is needed to understand the impact of genetic variations on stroke severity and to develop effective treatments.

*BDNF*: It is a crucial neurotrophic factor involved in neuronal survival, growth, and plasticity, playing a significant role in the central nervous system (CNS), influencing areas such as the hippocampus, cortex, and basal forebrain, which are vital for learning, memory, and higher thinking (Colucci‐D'amato et al. 2020; Oyovwi et al. 2025). BDNF is also involved in the regulation of glucose and energy metabolism (Bathina and Das 2015). The BDNF Val66Met polymorphism is a genetic variation involving a substitution of valine (Val) with methionine (Met) at position 66 in the BDNF gene, having been extensively studied for its implications in neurological and psychiatric disorders, with studies suggesting the Met allele may be associated with reduced BDNF secretion and impaired neuronal plasticity (Y. Zhang et al. 2024; French et al. 2018). This polymorphism has been linked to various neurological and psychiatric conditions, including neurodegenerative diseases like Alzheimer's disease (AD), Parkinson's disease, and multiple sclerosis, as well as psychiatric disorders such as major depressive disorder and generalized anxiety disorder (Colucci‐D'amato et al. 2020; Y. Zhang et al. 2024), with the Met allele associated with both protective and risk‐enhancing effects depending on the condition and context (Y. Zhang et al. 2024). While some studies have explored the role of the BDNF Val66Met polymorphism in stroke recovery, its association with initial stroke severity remains less clear and requires further investigation (French et al. 2018), with understanding this relationship potentially providing insights into potential therapeutic strategies for stroke patients.

*Antioxidant Genes*: Oxidative stress is a critical factor in the pathophysiology of stroke, especially ischemic stroke, which makes up about 80%–85% of all stroke cases (Allen and Bayraktutan 2009; Awooda and Awooda 2019). It occurs due to an imbalance between the production of reactive oxygen species (ROS) and the body's antioxidant defenses, resulting in cellular damage (Akinlua et al. 2019). In stroke, oxidative stress contributes to endothelial dysfunction, thrombosis, and neuronal injury (Awooda and Awooda 2019; Z. Li et al. 2022). Genetic variations in antioxidant enzymes like superoxide dismutase (SOD) and glutathione peroxidase (GPx) have been investigated for their potential in predicting stroke severity, but the findings have been inconsistent (Salminen and Paul 2014). Some studies indicate that genetic polymorphisms affecting these enzymes may influence susceptibility to oxidative stress‐related disorders (Salminen and Paul 2014). Variations in SOD and GPx genes have been linked to altered antioxidant activity, potentially impacting stroke outcomes (Salminen and Paul 2014). Further research is necessary to clarify the specific role of these genetic variations in stroke severity and to develop targeted therapies (Salminen and Paul 2014).

## 9 Genetic Predictors of Functional Outcome

Functional outcomes after stroke refer to the degree of disability and independence achieved by individuals’ poststroke, often assessed using tools like the mRS. The mRS is a widely used ordinal scale ranging from 0 (*no symptoms*) to 6 (*death*), measuring global disability and dependence in daily activities following a stroke. The scale includes: 0: *No symptoms*; 1: *No significant disability*; able to carry out usual activities despite some symptoms; 2: *Slight disability*; able to manage own affairs but unable to perform all previous activities; 3: *Moderate disability*; requires some help but can walk unassisted; 4: *Moderately severe disability*; needs assistance for bodily needs and cannot walk unassisted; 5: *Severe disability*; bedridden, incontinent, requiring constant care; and 6: *Death* (Harrison et al. 2013). Functional outcomes are influenced by age, stroke severity and type, where younger patients and those with moderate severity tend to recover better (Shin et al. 2022). Genetic factors affecting neuronal plasticity and recovery mechanisms may also play a role (Harrison et al. 2013). Rapid improvement typically occurs in the acute phase (up to 18 months), followed by a plateau or decline after 30 months (Harrison et al. 2013). Predicting functional outcomes is vital for tailoring rehabilitation strategies, setting realistic expectations, and optimizing long‐term care. Structured interviews or tools like the mRS‐SI improve reliability in assessing outcomes (Taylor‐Rowan et al. 2018).

### 9.1 Genes Involved in Neuroplasticity and Recovery

Neuroplasticity, the brain's ability to reorganize itself by forming new neural connections, is crucial for functional recovery after stroke. Genes involved in neurogenesis, synaptogenesis, and axonal remodeling have been investigated as potential predictors of functional outcome.

*BDNF*: It is crucial for neuronal plasticity and recovery after stroke, with the BDNF Val66Met polymorphism significantly impacting functional outcomes, as individuals with the Val/Val genotype may experience better recovery compared to Met allele carriers (X. Liu et al. 2021; Balkaya and Cho 2019; di Pino et al. 2016). BDNF is essential for neuroplasticity, the brain's ability to reorganize itself, which is important for stroke rehabilitation (X. Liu et al. 2021; di Pino et al. 2016). The Val66Met polymorphism affects BDNF release, with Val/Val individuals showing enhanced BDNF secretion and neuroplasticity compared to Met carriers, who may experience impaired plasticity and poorer functional outcomes (X. Liu et al. 2021; Balkaya and Cho 2019; di Pino et al. 2016). A meta‐analysis confirmed that the BDNF Val66Met polymorphism predicts functional outcomes poststroke, supporting that Val/Val individuals have a greater capacity for recovery (X. Liu et al. 2021; Parchure et al. 2022). Research indicates that recovery mechanisms may differ between genotypes, with Val/Val patients relying more on intracortical plastic processes and Met carriers potentially depending on subcortical mechanisms (Parchure et al. 2022). Some studies have reported mixed results regarding the impact of the Val66Met polymorphism on recovery, finding no significant differences in specific contexts such as poststroke aphasia, highlighting the complexity of genetic influences on stroke recovery (de Boer et al. 2017; Antal et al. 2010). Understanding BDNF's influence can inform personalized rehabilitation approaches, as exercise and other interventions can modulate BDNF levels, potentially benefiting those with specific genotypes (X. Liu et al. 2021; Balkaya and Cho 2019).

*Nogo Receptor 1 (NgR1)*: It limits axonal growth and plasticity by binding myelin‐associated inhibitors like Nogo‐A, upregulated after CNS injury (Petratos et al. 2020; Hirokawa et al. 2017) Blocking NgR1 enhances axonal regeneration and functional recovery in stroke models; for instance, NgR1 deletion in mice promotes cortical synaptic plasticity and axonal sprouting, and NEP1‐40 improves motor recovery when combined with therapies boosting neuronal growth (Akbik et al. 2013; X. Wang et al. 2012). NgR1's effects are linked to its regulation of paranodal domains and cytoskeletal stability, critical for axonal integrity (Petratos et al. 2020; Hirokawa et al. 2017). Human genetic studies on NgR1 variants and stroke outcomes are limited; the STRONG study focused on BDNF, COMT, and other stress‐related genes, but not NgR1 polymorphisms (Cramer et al. 2024). This highlights the need for targeted research on NgR1 gene variations, given its role in myelin plasticity and synaptic reorganization in animal models (Petratos et al. 2020; X. Wang et al. 2012). Current therapeutic strategies inhibit NgR1 signaling to disinhibit axonal growth, but clinical translation requires deeper insights into genetic and molecular interactions in human stroke populations (Hirokawa et al. 2017; X. Wang et al. 2012; Cramer et al. 2024).

*Dopamine‐Related Genes*: Dopamine is a neurotransmitter crucial for motor control, motivation, and reward, influencing movement by modulating sensitivity to the energetic cost of actions and enhancing motor vigor in response to rewards (Gepshtein et al. 2014; Michely et al. 2020). Dopamine also acts as a “teaching” signal, facilitating learning and operant conditioning by reinforcing behaviors that lead to rewards (Pearson‐Fuhrhop et al. 2013). Genetic variations in dopamine‐related genes, such as the dopamine transporter (DAT1) and dopamine receptors (DRD2, DRD3), have been studied for their impact on motor recovery and functional outcomes after stroke; some research suggests that specific DAT1 genotypes may be associated with better motor recovery (Bosun et al. 2024), though further confirmation is needed. DAT1 (dopamine transporter) regulates synaptic dopamine levels by reuptaking dopamine into presynaptic neurons, and variations in the DAT1 gene can affect dopamine neurotransmission and potentially influence motor learning and recovery (Pearson‐Fuhrhop et al. 2013). DRD2 (dopamine D2 receptor) is involved in reinforcement and reward processing, and variations, such as the A1/A2 allelic forms, can influence the number of receptors and affect dopaminergic function (Yadav et al. 2025). DRD3 (dopamine D3 receptor), found primarily in the ventral striatum, plays a role in motor control and reward processing (Pearson‐Fuhrhop et al. 2013).

### 9.2 Genes Involved in Inflammation and Immune Response

Inflammation in brain injury has a dual role, contributing to acute damage and facilitating later recovery. Resolution of inflammation and modulation of the immune response are critical for tissue repair and neuroplasticity. Genetic variations in key cytokines, like interleukin‐10 (IL‐10) and transforming growth factor‐beta 1 (TGF‐β1), may influence these processes and impact functional outcomes (Oyovwi and Udi 2025; Obukohwo, Oreoluwa, et al. 2024; Obukohwo, Joseph, et al. 2024; Postolache et al. 2020; Russo and McGavern 2016). IL‐10, an anti‐inflammatory cytokine, suppresses excessive inflammation and promotes tissue repair. Genetic variations in the IL‐10 promoter region have been studied for associations with stroke recovery; certain IL‐10 genotypes may favor better functional outcomes by balancing pro‐ and anti‐inflammatory responses. For example, it has been suggested that specific IL‐10 genotypes could enhance stroke recovery (Yin et al. 2015). TGF‐β1, a pleiotropic cytokine involved in inflammation, fibrosis, and wound healing, has genetic variations that have been investigated in stroke recovery, though results are inconsistent. While TGF‐β1 has potential roles in modulating inflammation and promoting repair, further research is needed to clarify its genetic influence on recovery outcomes (Postolache et al. 2020; Russo and McGavern 2016). In traumatic brain injury (TBI), inflammation is also a double‐edged sword: acute neuroinflammation aids tissue regeneration and debris clearance, but chronic inflammation can lead to neurodegeneration. Modulating the immune response, rather than suppressing it entirely, may optimize recovery. Studies highlight the importance of balancing pro‐inflammatory and reparative immune activation, with genetic factors playing a role in this balance (Postolache et al. 2020; Russo and McGavern 2016).

### 9.3 Genes Involved in Angiogenesis

Angiogenesis, the formation of new blood vessels, is a critical process in tissue repair and recovery following ischemic stroke. It restores blood flow to the ischemic penumbra, promotes neuronal regeneration, and supports functional recovery. Enhanced vascular endothelial growth factor (VEGF) expression has been linked to improved outcomes after ischemic stroke by promoting neovascularization in the ischemic penumbra and supporting neurogenesis and synaptic plasticity (Yin et al. 2015; Hu et al. 2024). Angiogenesis begins within 24 h poststroke and can persist for weeks to months. This process is crucial for repairing the cerebrovascular system (Yin et al. 2015). VEGF interacts with other factors such as fibroblast growth factors (FGFs), matrix metalloproteinases (MMPs), and inflammatory cytokines to regulate endothelial cell behavior during vessel formation (Shi et al. 2023). Inflammatory cells like macrophages release VEGF and other angiogenic factors, contributing to endothelial sprouting and vessel stabilization (Shi et al. 2023). Certain genetic polymorphisms in the VEGF promoter region may influence its expression and activity. Studies suggest that specific VEGF genotypes are associated with better angiogenesis and improved functional recovery after stroke (Yin et al. 2015; Zhu et al. 2021). However, the precise mechanisms by which these genetic variations affect VEGF expression and angiogenic efficiency remain under investigation. The density of new microvessels in the penumbra correlates with improved survival rates and neurological recovery in both animal models and human patients (Hu et al. 2024; Zhu et al. 2021). Enhancing angiogenesis through therapeutic strategies targeting VEGF and related pathways holds promise for improving stroke outcomes. However, challenges remain in optimizing these therapies to balance angiogenesis with potential risks like abnormal vascular growth or hemorrhage.

## 10 Genetic Predictors of Poststroke Depression

Poststroke depression (PSD) is a common and debilitating complication, affecting about one‐third of stroke patients and significantly impacting recovery, leading to poorer functional outcomes, increased mortality, and reduced rehabilitation adherence (Nickel and Thomalla 2017; Capaldi and Wynn 2010; Paolucci et al. 2019). While environmental factors like social isolation and functional disability play a role, genetic vulnerability is also critical in PSD development. The SLC6A4 gene, encoding the serotonin transporter (SERT), regulates serotonin levels, and variations like the 5‐HTTLPR polymorphism are linked to depression; the short (S) allele may increase PSD risk, as shown in a Korean population (J. M. Kim et al. 2013; Kang et al. 2021), though findings vary, necessitating larger studies (J. M. Kim et al. 2013; Lam et al. 2018). The BDNF gene affects neuronal survival and plasticity, and the Val66Met polymorphism (rs6265) is implicated in depression; the Met allele, associated with impaired BDNF secretion and neuroplasticity, may increase PSD risk, as confirmed by a meta‐analysis (Nickel and Thomalla 2017), though conflicting results exist. Stroke‐induced inflammation contributes to neuronal damage and PSD, and genetic variations in inflammatory cytokine genes like IL‐1β, IL‐6, and TNF‐α have been linked to depression; polymorphisms in IL‐1β were associated with PSD risk, while TNF‐α variations correlated with depressive symptoms in cardiovascular patients (Nickel and Thomalla 2017; Paolucci et al. 2019). Other genes involved in HPA axis regulation (NR3C1) and dopamine signaling (SLC6A3) are being investigated, although evidence is preliminary (Nickel and Thomalla 2017). Understanding the genetic basis of PSD can help identify at‐risk patients for targeted interventions like antidepressant therapy or personalized rehabilitation (Capaldi and Wynn 2010; Paolucci et al. 2019), and further research into genetic markers and their interactions with environmental factors is essential to improve PSD management.

### 10.1 Genetic Predictors of Poststroke Cognitive Impairment

Poststroke cognitive impairment (PSCI) is a common and debilitating consequence of stroke, affecting a significant proportion of stroke survivors and contributing to long‐term disability and dementia (El Husseini et al. 2023). PSCI encompasses a range of cognitive deficits, including memory impairment, executive dysfunction, and language difficulties. While stroke severity and lesion location are important determinants of PSCI, genetic factors are increasingly recognized as contributing to individual vulnerability.

The APOE gene is a key genetic factor influencing the risk of AD and PSCI. The APOE gene encodes APOE, a protein involved in lipid transport and neuronal repair. It has three common alleles: ε2, ε3, and ε4. The ε4 allele is associated with an increased risk of AD and PSCI, promoting amyloid deposition, oxidative stress, and impaired neuronal repair mechanisms (C. C. Liu et al. 2013; J. Kim et al. 2009) Individuals carrying one ε4 allele have a 2–3‐fold increased risk of late‐onset AD, while those with two alleles face a 10–15‐fold higher risk (J. Kim et al. 2009; Yamazaki et al. 2019), potentially through accelerated amyloid‐beta (Aβ) accumulation and aggregation (Yamazaki et al. 2019; C. C. Liu et al. 2013), increased tau pathology and neuroinflammation (J. Kim et al. 2009; Yamazaki et al. 2019), and impaired synaptic plasticity and mitochondrial dysfunction (J. Kim et al. 2009). Studies suggest that the ε4 allele may also heighten the risk of cognitive decline after stroke, particularly lacunar stroke, potentially via amyloid deposition, oxidative stress, and reduced neuronal repair (C. C. Liu et al. 2013), though findings on its association with PSCI are inconsistent, likely due to interactions with other genetic or environmental factors (C. C. Liu et al. 2013). The ε2 allele offers protective effects against AD but may not prevent amyloid deposition in advanced age (Yamazaki et al. 2019; C. C. Liu et al. 2013), while the ε3 allele is the most common and considered neutral regarding AD risk (J. Kim et al. 2009). Research into APOE‐targeted therapies aims to address its role in Aβ clearance, neuroinflammation, and synaptic dysfunction, with strategies including modifying APOE expression or isoform‐specific functions to mitigate its pathological effects in AD and potentially PSCI (J. Kim et al. 2009; Raulin et al. 2022).

VEGF is a potent angiogenic factor critical in neurovascular remodeling and stroke recovery, with genetic variations influencing its expression and potentially impacting neurovascular repair and poststroke cognitive outcomes. VEGF promotes angiogenesis and neurogenesis, essential for functional recovery after ischemic injury; it is released naturally by endothelial cells poststroke and can be enhanced through exogenous administration or therapies like mesenchymal stem cell transplantation (C. Liu et al. 2023; Talwar and Srivastava 2014). VEGF‐A, the most studied isoform, mobilizes endothelial progenitor cells (EPCs) crucial for vascular repair, with higher circulating EPC levels poststroke correlating with better recovery outcomes (Greenberg and Jin 2013). Polymorphisms in the VEGF gene have been linked to reduced angiogenesis and poorer cognitive recovery after stroke, suggesting that genetic differences affecting VEGF expression may contribute to PSCI (Talwar and Srivastava 2014; Shim and Madsen 2018). VEGF signaling pathways, particularly involving VEGFR‐2, play a significant role in regulating neurovascular repair mechanisms, and alterations in these pathways due to genetic variations could exacerbate stroke‐induced damage (Shim and Madsen 2018; Shibuya 2011). Enhancing VEGF activity through targeted therapies, such as stem cell transplantation or physical interventions, has shown promise in improving neurovascular remodeling and reducing infarct volume (C. Liu et al. 2023; Talwar and Srivastava 2014). Continuous delivery of VEGF or strategies to prolong its biological half‐life could be beneficial for stroke treatment, though challenges remain (C. Liu et al. 2023). These findings underscore the importance of VEGF in stroke recovery and highlight the potential impact of genetic variations on therapeutic outcomes.

The ACE gene is central to the renin‐angiotensin system (RAS), which is vital for blood pressure regulation and vascular function. A key polymorphism in the ACE gene is the insertion/deletion (I/D) polymorphism, characterized by the presence or absence of a 287‐bp Alu sequence in intron 16; this affects ACE activity, with the DD genotype linked to higher ACE activity and the II genotype to lower activity (Shibuya 2011; Nouryazdan et al. 2022; Thakur et al. 2022). The ACE I/D polymorphism has been associated with an increased risk of cardiovascular diseases, including atherosclerosis and heart failure; higher ACE levels, often related to the DD genotype, can increase angiotensin II production, promoting vasoconstriction and sodium retention, contributing to hypertension and cardiovascular disease progression (Shibuya 2011; Nouryazdan et al. 2022; Niu et al. 2002). Studies indicate that the DD genotype is associated with increased hypertension risk across populations; increased ACE activity in DD genotype individuals can lead to higher blood pressure due to enhanced vasoconstriction and fluid retention (Nouryazdan et al. 2022; Bonney et al. 2024). Furthermore, the DD genotype has been linked to pregnancy complications such as preeclampsia and intrauterine growth restriction (IUGR), likely due to impaired placentation and altered vascular function (Shibuya 2011). Evidence suggests the DD genotype may contribute to vascular dementia by promoting vascular damage and impairing cerebral blood flow, potentially increasing the risk of PSCI (Rost et al. 2022).

Other candidate genes that have been investigated in relation to PSCI include those involved in neuroinflammation, oxidative stress, and synaptic plasticity. For example, polymorphisms in genes encoding inflammatory cytokines, such as IL‐6, and antioxidant enzymes, such as SOD, have been associated with cognitive decline after stroke (Ołdakowska et al. 2022; Vogrinc et al. 2023). However, more research is needed to confirm these associations and identify other genetic markers that contribute to PSCI.

### 10.2 Genetic Predictors of Poststroke Seizures

Poststroke seizures (PSS) are a relatively common complication following stroke, affecting approximately 5%–10% of stroke survivors (J. Zhou et al. 2023). PSS can have a significant impact on patient recovery and quality of life, increasing the risk of further neurological damage and interfering with rehabilitation efforts. While stroke severity and lesion location are important risk factors for PSS, genetic predisposition is also thought to play a role.

Genetic factors influence PSS, often classified as poststroke epilepsy (PSE). These genes are primarily associated with epilepsy‐related mechanisms such as ion channel function, inflammation, neurotransmitter signaling, and synaptic plasticity. Polymorphisms in voltage‐gated sodium channel genes, such as SCN1A and SCN2A, which play a critical role in neuronal excitability, are frequently mutated in epilepsy and have been explored as potential risk factors for PSS, though evidence linking them directly to PSS remains limited (Altman et al. 2019; J. Wang et al. 2017). Other ion channels, including calcium and potassium channels, may also contribute to epileptogenesis through aberrant activity (Shevlyakov et al. 2023). Dysregulation of glutamate signaling is central to seizure susceptibility, and genes encoding glutamate receptors, such as GRIA1 and GRIN2A, have been associated with epilepsy; their role in excitatory neurotransmission suggests potential involvement in PSS, although no direct studies link these genes to PSS (Altman et al. 2019; J. Wang et al. 2017). Genes encoding GABA receptors (GABRA1, GABRB3), which are critical for inhibitory signaling in the brain, may disrupt the balance between excitation and inhibition, contributing to seizure development if variations are present (Shevlyakov et al. 2023). Genetic variations in inflammatory cytokine genes, such as IL‐1β and TNF‐α, have been linked to an increased risk of early‐onset PSS, as inflammation following a stroke lowers the seizure threshold by disrupting neuronal excitability; for instance, IL‐1β polymorphisms promote neuronal hyperexcitability through enhanced inflammatory responses (Altman et al. 2019; J. Wang et al. 2017). Synaptic proteins involved in plasticity, such as SYN1 and SYN2, are candidate genes for PSS, regulating synaptic remodeling and neuronal connectivity, processes that can become maladaptive after a stroke (Altman et al. 2019; J. Wang et al. 2017). Stroke‐induced BBB breakdown allows blood components like thrombin to enter the brain parenchyma, activating protease‐activated receptor 1 (PAR1), enhancing NMDA receptor activity and calcium influx, leading to hyperexcitability and maladaptive plasticity (Altman et al. 2019). Stroke triggers intense neuroinflammation mediated by cytokines like IL‐1β and TNF‐α, disrupting inhibitory synapses and promoting synchronous neuronal firing (Shevlyakov et al. 2023). Excessive glutamate release during ischemia drives excitotoxicity, contributing to epileptic focus formation through long‐term potentiation (LTP) and maladaptive plasticity (Altman et al. 2019).

### 10.3 Genetic Predictors of Poststroke Spasticity

Spasticity, characterized by increased muscle tone and exaggerated reflexes, is a common complication following stroke (Kuo and Hu 2018). Spasticity can impair motor function, cause pain, and interfere with rehabilitation efforts. While the pathophysiology of spasticity is complex and involves both central and peripheral mechanisms, genetic factors are thought to contribute to individual susceptibility.

Collagen, a major structural protein in connective tissue, plays a crucial role in maintaining muscle tone and flexibility. Genetic variations in collagen genes, such as COL1A1 and COL3A1, have been associated with altered collagen synthesis and tissue elasticity. Even though it has been indicated that there is a possible role of collagen related genes in the spasticity, the direct association between COL1A1 and post stroke spasticity is speculative and requires further studies to establish any association. A study by Collins and Raleigh (2009) found that certain COL1A1 gene polymorphisms were associated with increased muscle stiffness and susceptibility to musculoskeletal injuries. While no specific study has directly linked COL1A1 polymorphisms with poststroke spasticity, the potential link between collagen structure and muscle tone suggests that these genes may play a role in spasticity.

Dopamine, a neurotransmitter involved in motor control, plays a role in the regulation of muscle tone and movement. Genetic variations in dopamine receptor genes, such as DRD2 and DRD4, have been associated with altered dopamine signaling and movement disorders. A study by Magistrelli et al. (2021) found that certain DRD2 gene polymorphisms were associated with increased risk of Parkinson's disease, a neurodegenerative disorder characterized by rigidity and tremor. While no specific study has directly linked DRD2 polymorphisms with poststroke spasticity, the potential link between dopamine signaling and motor control suggests that these genes may play a role in spasticity.

Neurotrophic factors, such as BDNF, play a critical role in neuronal survival, growth, and plasticity. They are also involved in the regulation of motor neuron function and muscle innervation. Genetic variations in neurotrophic factor genes, such as GDNF (Glial cell line‐derived neurotrophic factor), have been associated with altered motor neuron function and muscle strength. Previous reports have shown that certain GDNF gene polymorphisms were associated with increased susceptibility to amyotrophic lateral sclerosis (ALS), a neurodegenerative disease characterized by motor neuron degeneration and muscle weakness (Ovsepian et al. 2023). While no specific study has directly linked GDNF polymorphisms with poststroke spasticity, the potential link between neurotrophic factors and motor neuron function suggests that these genes may play a role in spasticity.

Other candidate genes that have been investigated in relation to spasticity include those involved in muscle contraction, such as the actin genes (ACTA1, ACTG1), and those involved in neuronal excitability, such as the potassium channel genes (KCNQ2, KCNQ3) (Ovsepian et al. 2023). However, the evidence for these associations is still preliminary, and further research is needed to confirm their role in spasticity. However, the effects of other factors, such as rehabilitation therapy, physical activity and diets have been shown to modulate genetic effects on PSS and or spasticity. For instance, a previous study has shown that rehabilitation therapy reduces spasticity improving chloride ion homeostasis by restoring the BDNF‐KCC2 pathways, a Cl^−^ extruder which contribute to the development of spasticity in motoneurons following spinal cord injury in rats by enhancing Cl^−^ extrusion and hyperpolarization (Beverungen et al. 2020).

## 11 Genetic Influences on Stroke Recovery and Rehabilitation

The field of rehabilitation genomics seeks to understand how an individual's genetic makeup influences their response to rehabilitation interventions poststroke (Beverungen et al. 2020). This knowledge holds immense potential for personalized rehabilitation strategies, allowing clinicians to tailor therapies based on a patient's genetic profile, thereby maximizing their recovery potential. Several candidate genes and genetic variants have been investigated for their association with stroke recovery, particularly in the context of motor function, language, and cognitive rehabilitation.

### 11.1 Genes Involved in Neuroplasticity and Synaptic Remodeling

The BDNF Val66Met polymorphism influences stroke recovery via neuroplasticity and rehabilitation outcomes. BDNF is crucial for neuronal survival, growth, differentiation, and synaptic plasticity, supporting LTP (Balkaya and Cho 2019; de Boer et al. 2017). The rs6265 (Val66Met) SNP in the BDNF gene affects activity‐dependent BDNF secretion, with the Val allele associated with enhanced BDNF secretion and greater neuroplasticity, while the Met allele is linked to reduced BDNF secretion and dampened cortical plasticity (Balkaya and Cho 2019; de Boer et al. 2017). Studies suggest Val/Val genotype stroke patients show superior motor recovery during intensive rehabilitation (French et al. 2018), though conflicting results exist (French et al. 2018). Research indicates the Met allele may negatively affect functional mobility in chronic stroke survivors (French et al. 2018), but its influence on language recovery is unclear (Suman et al. 2024). Some studies suggest Met/Met carriers may show enhanced motor recovery in the chronic phase due to compensatory mechanisms (Zedde et al. 2025). Variability in findings is attributed to lesion characteristics, rehabilitation protocols, and demographics (Aderinto et al. 2023).

NgR1 is a neuronal receptor that inhibits axonal growth and plasticity, particularly in the CNS. Blocking NgR1 signaling has been shown to promote axonal regeneration and functional recovery in animal models of stroke (Khan and Nasir 2024). Genetic variations in the *RTN4R* gene, which encodes NgR1, may influence the degree of axonal sprouting and plasticity following stroke. Studies have explored the association between *RTN4R* polymorphisms and motor recovery, with some evidence suggesting that certain haplotypes are associated with improved functional outcomes (Giordano et al. 2022). For example, Santoro et al. (2020) found that a specific *RTN4R* haplotype was associated with increased independence in activities of daily living (ADL) in stroke patients undergoing rehabilitation.

Dopamine, a neurotransmitter crucial for motor control, motivation, and reward, plays a significant role in motor learning and rehabilitation. Genetic variations in genes involved in dopamine synthesis, transport, and metabolism can influence the effectiveness of rehabilitation therapies. The COMT gene encodes catechol‐*O*‐methyltransferase, involved in dopamine metabolism; Val/Val, Val/Met, and Met/Met polymorphisms have been associated with motor recovery after stroke, with the Val/Val polymorphism often linked to better recovery (Bradley and Damiano 2019; B. R. Kim et al. 2016). The DRD1 and DRD3 genes encode dopamine receptors D1 and D3, respectively, and may influence motor learning and rehabilitation outcomes (Bradley and Damiano 2019; B. R. Kim et al. 2016). The DRD2 gene encodes the dopamine D2 receptor; the DRD2 TaqIA polymorphism affects dopamine receptor density and function, with the A1 allele associated with lower D2 receptor availability; some studies suggest this allele is linked to poorer motor outcomes after stroke, though results are inconsistent. SLC6A3 (dopamine transporter) warrants further investigation for its potential influence on rehabilitation outcomes. Individuals with higher dopamine gene scores may perform better in motor learning tasks without additional dopamine, while those with lower scores may benefit from dopaminergic medications like l‐Dopa (Collins and Raleigh 2009). Using rewards to enhance dopamine levels may improve learning outcomes in individuals with lower dopamine gene scores (Dryden et al. 2025).

### 11.2 Genes Involved in Inflammatory and Immune Responses

Genetic variations in genes regulating inflammation and immune responses can influence the balance between detrimental and beneficial inflammatory effects poststroke, impacting recovery outcomes; this interplay between genetics, inflammation, and stroke recovery has been extensively researched (Nikolic et al. 2020). IL‐6, a pro‐inflammatory cytokine crucial in acute stroke pathology and long‐term recovery, shows that polymorphisms in the IL‐6 gene have been associated with variations in IL‐6 levels and inflammatory responses, potentially affecting stroke recovery; for example, the G allele of the IL‐6‐174G/C polymorphism has been linked to poorer motor recovery at 3 months poststroke compared to the C/C genotype; however, the impact of IL‐6 polymorphisms on stroke recovery may depend on various factors (Nikolic et al. 2020). TNF‐α, another critical pro‐inflammatory cytokine in the poststroke inflammatory cascade, shows that genetic polymorphisms in the TNF gene have been associated with variations in TNF‐α levels and inflammatory responses; some studies suggest that certain TNF genotypes are associated with an increased risk of poor functional outcomes after stroke, but the relationship between TNF polymorphisms and stroke recovery is not fully understood and requires further investigation (Nikolic et al. 2020). Recent GWAS have identified variants in the PATJ and LOC genes as potential genetic markers for long‐term stroke outcomes (Nikolic et al. 2020). Epigenetic mechanisms, including histone modifications, DNA methylation, and noncoding RNA activity, also play a role in regulating the immune response after stroke, contributing to inflammation and recovery (Nikolic et al. 2020). Understanding the genetic aspects of inflammation in stroke recovery has implications for developing personalized treatment strategies, where pharmacogenetics and pharmacogenomics of stroke treatments could be evaluated in the context of immuno‐inflammation and brain plasticity, and potential novel genetic treatment modalities are being explored, although they are still in the early stages of investigation (Nikolic et al. 2020).

### 11.3 Genes Involved in Angiogenesis and Vascular Remodeling

Angiogenesis, the formation of new blood vessels, is crucial for tissue repair and recovery after ischemic stroke by restoring blood flow, delivering oxygen and nutrients, and promoting neuronal regeneration. Angiogenic processes begin within 24 h of stroke onset and persist for weeks to months, enhancing microvascular density in peri‐infarct regions (Hu et al. 2024). This process stabilizes brain perfusion, aiding neuronal survival and brain plasticity, supporting neurogenesis and synaptic remodeling, and contributing to functional recovery (Hu et al. 2024). Angiogenesis involves the neurovascular unit, including endothelial cells, pericytes, and signaling pathways such as VEGF‐mediated mechanisms (Simon et al. 2017). VEGF is a potent pro‐angiogenic factor that promotes endothelial cell proliferation and migration. Genetic polymorphisms in the VEGF gene influence angiogenic responses; for example, the VEGF+405G/C polymorphism has been associated with improved functional outcomes in stroke patients carrying the C allele (Ergul et al. 2012). However, therapeutic angiogenesis faces challenges. Newly formed vessels may have increased permeability, risking edema, and hemorrhagic transformation (Yang and Torbey 2020). Inflammatory signaling can influence endothelial cell behavior but may also exacerbate damage if dysregulated (Zhu et al. 2021). Premorbid conditions like diabetes or hypertension can impair angiogenic responses and functional outcomes (Ergul et al. 2012). Future research aims to optimize angiogenic therapies by balancing vessel stability and functional restoration while mitigating risks like barrier dysfunction (Yang and Torbey 2020). Genetic studies on VEGF polymorphisms could inform personalized therapeutic strategies to enhance stroke recovery (Navaratna et al. 2009).

## 12 Genetic Predictors of Stroke Recurrence

Stroke recurrence is a significant concern, with approximately 25%–40% of stroke survivors experiencing another stroke within five years (B. Lin et al. 2021). Identifying individuals at high risk for stroke recurrence is crucial for implementing preventive strategies and improving long‐term outcomes. Several genetic factors have been implicated in influencing the risk of stroke recurrence, particularly those related to vascular function, blood coagulation, and lipid metabolism.

### 12.1 Genes Involved in Vascular Function and Structure

The NOS3 Glu298Asp polymorphism (rs1799983) influences cardiovascular pathophysiology and stroke risk by affecting endothelial nitric oxide synthase (eNOS) function; the G allele homozygote (GG genotype) is significantly associated with brain infarction, especially lacunar stroke (OR 1.56–2.00) (Elbaz et al. 2000), correlating with reduced NO bioavailability, impaired vasodilation, and increased endothelial dysfunction (Oliveira‐Paula et al. 2016). In stroke cases, the GG genotype frequency was 46.1% versus 35.4% in controls, with a stronger association in lacunar subtypes (OR 2.00) (Elbaz et al. 2000; Thakur et al. 2022), aligning with small‐vessel arteriolopathy from NO deficiency; synergistic effects exist between the GG genotype and elevated LDL cholesterol, suggesting combined genetic and metabolic pathways in stroke pathogenesis (Elbaz et al. 2000). Some studies found no association with stroke recurrence (Yu et al. 2006), while meta‐analyses in Asian populations indicate increased coronary heart disease risk (OR 1.12–1.59) (Tian et al. 2013), highlighting population‐specific genetic influences. eNOS dysfunction from the Glu298Asp variant may lead to reduced NO‐mediated vasodilation, increased oxidative stress due to superoxide production (Tran et al. 2022), and enhanced platelet aggregation and leukocyte adhesion (Oliveira‐Paula et al. 2016). Subtype‐specific effects (stronger links to lacunar vs. atherothrombotic stroke) (Elbaz et al. 2000), ethnic variability in allele frequencies and risk profiles (Tian et al. 2013), and interactions with cofactors like BH4 and environmental factors (e.g., LDL) (Elbaz et al. 2000; Oliveira‐Paula et al. 2016) are limitations and confounders; further studies integrating NOS3 haplotypes, epigenetic regulators, and proteolytic enzymes like MMP‐9 are needed to clarify their combined roles in stroke recurrence risk.

### 12.2 Genes Involved in Blood Coagulation

FVL, caused by a mutation in the F5 gene, and the prothrombin G20210A mutation are genetic variants that primarily increase the risk of VTE, including DVT and PE, and have also been studied for their potential roles in arterial thrombosis, including stroke. FVL leads to a protein that resists inactivation by APC, thus increasing blood clotting propensity (Kujovich 2011), and is the most common inherited risk factor for VTE, affecting about 20%–25% of patients with VTE (Kujovich 2011). While primarily associated with venous thrombosis, FVL has been linked to an increased risk of ischemic stroke, particularly in young adults and those with cryptogenic strokes (Hamedani et al. 2010), and is also associated with a higher risk of pregnancy loss and other obstetric complications (Kujovich 2011). The prothrombin G20210A mutation affects the prothrombin gene, increasing prothrombin levels and thus enhancing blood clotting, increasing the risk of VTE, similar to FVL. Evidence suggests its involvement in arterial thrombosis, including stroke, particularly in younger individuals and those with cryptogenic strokes (Chiasakul et al. 2019); however, like FVL, the association with recurrent stroke is not conclusively established, and routine screening is not recommended for all stroke patients.

### 12.3 Genes Involved in Lipid Metabolism

APOE is a multifunctional 299 amino acid protein (approximately 34 kDa) crucial for lipid metabolism, neurobiology, and the pathogenesis of neurodegenerative diseases; it exists as part of several classes of lipoprotein particles, including chylomicron remnants, VLDL, IDL, and some HDL (C. C. Liu et al. 2013). The APOE gene encodes APOE and has three major alleles: APOE2, APOE3, and APOE4, which influence lipid transport and metabolism differently, impacting various physiological and pathological processes (Y. Huang and Mahley 2014). APOE plays a central role in lipid transport by binding to cell‐surface receptors, facilitating the uptake of lipoproteins by cells to maintain cholesterol homeostasis (C. C. Liu et al. 2013; Y. Huang and Mahley 2014). The APOE2 isoform is associated with impaired receptor binding, leading to Type III hyperlipoproteinemia and higher triglyceride levels (Y. Huang and Mahley 2014). APOE3 is the most common, considered the “normal” form, and efficiently binds to LDL receptors, facilitating lipid metabolism (Y. Huang and Mahley 2014). APOE4 is associated with an increased risk of AD and cardiovascular diseases, including atherosclerosis and stroke, and is linked to higher LDL‐cholesterol levels and impaired lipid metabolism (Husain et al. 2021). Specifically, the APOE4 allele is a significant risk factor for late‐onset AD, influencing amyloid‐β aggregation and clearance, neuroinflammation, and tau pathology (Husain et al. 2021; Yamazaki et al. 2019). Research is ongoing to target APOE for therapeutic strategies in AD, focusing on modulating APOE quantity, lipidation, and interaction with amyloid‐β (Yamazaki et al. 2019).

## 13 Emerging Therapies Targeting Genetic Pathways

New treatment interventions that can be used to address genetic pathways provide a potential opportunity in preventing the recurrence of strokes, especially among genetically susceptible individuals. Nitric oxide (NO) is a major mediator of vasodilation and anti‐inflammatory actions whose synthesis is controlled by the NOS3 gene that encodes eNOS (Kidd et al. 2005). NOS3 variants including the Glu298Asp polymorphism has been reported to be related to low NO bioavailability, endothelial dysfunction, and the risk of stroke (OR = 1.22, 95% CI: 1.101.35) (Malik et al. 2018). Therapies that augment eNOS activity have been studied preclinically including tetrahydrobiopterin (BH4) supplementation, which has been shown to restore NO production and improve vascular function in animal models of stroke and decrease infarct size by about 30% (L. L. Tang and Zheng 2011).

The APOE gene that affects lipid metabolism and neuroinflammation is another promising target. APOE 4, found in about 20% of the population, is linked with increased LDL cholesterol levels, atherosclerosis, and worse poststroke cognitive (Haan and Mayeda 2010; Koizumi et al. 2018). Therapeutic approaches against APOE are antisense oligonucleotides (ASOs), that lower APOE expression, and have been found to be effective in lowering amyloid burden in AD models, and may be applicable to stroke given the similar pathological mechanisms (Huynh et al. 2017). Also, the effects of PCSK9 inhibitor like evolocumab have been shown to indirectly alter APOE pathways through LDL cholesterol reduction which has been found to reduce the risk of recurrent stroke (Giugliano et al. 2020). These treatments have hope of patients at risk of APOE‐related stroke, especially those with small vessel disease or cognitive impairment after stroke. Further development of such genetically informed treatments is vital in order to confirm their effectiveness and implement them into the clinical setting.

## 14 Challenges and Future Directions

### 14.1 Challenges

Identifying and validating genetic factors influencing stroke risk and outcome presents a formidable array of challenges. Stroke, a highly heterogeneous condition, arises from diverse etiologies including large artery atherosclerosis, small vessel disease, cardioembolism, and other less common causes (Murphy and Werring 2020). This inherent heterogeneity complicates genetic studies, as different genetic variants may contribute to specific stroke subtypes, demanding large, well‐phenotyped cohorts to dissect these nuances. Furthermore, the genetic architecture underlying stroke is likely complex, involving numerous common variants each with a small effect size, along with potentially rare variants with larger impacts. Disentangling these intricate interactions and identifying the truly causal variants amidst a sea of background noise requires sophisticated analytical methods and substantial statistical power.

Environmental influences play a significant role in stroke risk, further complicating the picture. Lifestyle factors like diet, smoking, and physical activity, as well as comorbidities like hypertension and diabetes, interact with genetic predispositions, making it challenging to isolate the independent impact of genetic variants (Gropper 2024). Accounting for these environmental factors and their interactions with genes requires comprehensive data collection and complex statistical modeling. Many genetic studies are also hampered by limited sample sizes, particularly when investigating specific stroke subtypes or populations (Dichgans and Markus 2005). This lack of statistical power diminishes the ability to detect true associations and increases the likelihood of false positive findings. Moreover, the difficulty in replicating initial findings across independent cohorts further underscores the challenges of accurately identifying and validating genuine genetic risk factors.

Current genetic studies also suffer from several limitations. A significant bias toward European populations exists, hindering the generalizability of findings to other ethnicities with potentially different genetic backgrounds and environmental exposures (Cerdeña et al. 2022). This lack of diversity limits our understanding of the global genetic landscape of stroke and perpetuates health disparities. Furthermore, many studies primarily focus on common genetic variants identified through GWAS, potentially overlooking the contribution of rare variants, which, although individually infrequent, may collectively account for a substantial portion of the heritability of stroke. Investigating these rare variants necessitates large‐scale sequencing efforts and advanced analytical techniques. Finally, a critical challenge lies in translating genetic findings into tangible clinical benefits. While identifying genetic risk factors is a crucial first step, understanding their functional consequences and developing targeted interventions based on these insights remains a significant hurdle. The pathway from genetic discovery to effective prevention and treatment strategies for stroke is often long and arduous, requiring multidisciplinary collaborations and innovative approaches.

### 14.2 Future Directions

Advancing our understanding of stroke genetics and translating this knowledge into improved clinical outcomes requires a multi‐faceted approach focused on expanding the scope and depth of our research. The key to the development of stroke genetics research and its application to clinical practice is multidisciplinary collaborations. These collaborations address complex challenges by leveraging the knowledge of geneticists, clinicians, epidemiologists, bioinformaticians, and ethicists. Future studies must prioritize the execution of larger and more diverse GWAS and subsequent meta‐analyses (Kujovich 2011; Hamedani et al. 2010; Tsalta‐Mladenov et al. 2022). Broadening the representation across different ancestral populations is crucial to uncover population‐specific genetic risk factors and enhance the generalizability of findings. In addition to GWAS, the application of advanced genomic technologies such as whole‐exome sequencing (WES) and whole‐genome sequencing (WGS) holds immense promise for identifying rare and novel genetic variants that may contribute significantly to stroke risk or outcome, particularly in individuals without common risk alleles (Lee et al. 2025; Yoshimoto et al. 2025; Ikram et al. 2009; Franco et al. 2011).

Furthermore, a holistic approach is needed, integrating genetic data with other “omics” data, including proteomics, metabolomics, and transcriptomics (Victor Oluwaloseyi et al. 2024). This systems‐level approach will provide a more comprehensive understanding of stroke pathophysiology, moving beyond individual genes to elucidate complex biological pathways and networks. Identified genetic variants require rigorous validation through functional studies, including cellular and animal models, to confirm their roles in stroke development and progression and to elucidate their underlying mechanisms of action.

The ultimate goal is to leverage this knowledge for precision medicine approaches in stroke prevention and treatment. Individual genetic profiles can be utilized to personalize risk assessment, tailor preventive strategies, and optimize therapeutic interventions. The development and refinement of polygenic risk scores (PRS) for stroke risk prediction are crucial steps in this direction, enabling the identification of individuals at high risk who could benefit from targeted interventions. However, the implementation of genetic testing for stroke risk and outcome necessitates careful consideration of ethical considerations, including genetic counseling, privacy, and the potential for discrimination. Responsible and equitable application of these technologies will be paramount to ensure that the benefits of stroke genetics research are realized for all.

## 15 Limitations of the Study

The review only searches PubMed, Embase, and Web of Science, so it may miss other relevant studies from databases like Cochrane Library or gray literature, which could provide new insights. It includes only English‐language studies, potentially introducing bias by excluding significant research published in other languages. Additionally, it filters out smaller studies, which could omit rare genetic information. To ensure methodological rigor, the review excludes studies with small sample sizes (e.g., fewer than 50 participants for case‐control studies or fewer than 100 for prospective studies). While this improves the quality of included studies, it may exclude data on rare genetic variants or specific populations where large samples are impractical. The review mainly focuses on common genetic variants identified through GWAS, possibly missing rare variants that also significantly influence stroke heritability. Detecting these rare variants requires extensive sequencing, which is not covered in the included studies.

## 16 Conclusion

This review has synthesized the current understanding of the intricate relationship between genetics and stroke, highlighting key genetic variants and pathways implicated in both stroke risk and outcome. The evidence presented underscores the undeniable importance of genetics in deciphering the complex etiology of stroke. Genetic factors contribute significantly to an individual's susceptibility to stroke, influencing various aspects from vascular health to inflammation and clotting mechanisms. Moreover, genetic makeup appears to play a crucial role in determining an individual's response to treatment and their overall recovery trajectory following a stroke event. Looking ahead, the ongoing advancements in genetic research hold immense promise for revolutionizing stroke care. Moreover, the knowledge gained from stroke genetics enhances stroke management, reduces patient suffering, and advances the healthcare system, notably increasing the real‐world impact based on developments in stroke genetics. By identifying individuals at high genetic risk, preventative strategies can be tailored to minimize their likelihood of experiencing a stroke. Additionally, genetic biomarkers could improve stroke diagnosis speed and accuracy, allowing for timely treatment. Pharmacogenomics offers personalized medicine based on genetics. However, our knowledge of stroke's genetic landscape is incomplete, with complex GxE interactions and rare variants needing further study. Future research should include large, multi‐ethnic studies to validate findings and discover new genetic targets. Continued investment in genetic research is vital to unlock personalized medicine's full potential and combat stroke.

## Author Contributions

**Mega Obukohwo Oyovwi**: conceptualization, investigation, writing–original draft, methodology, validation, writing–review and editing, project administration, supervision, resources. **Benneth Ben‐Azu**: conceptualization, investigation, writing–original draft, methodology, validation, writing–review and editing, resources, supervision, project administration. **Ejayeta Jeroh**: investigation, methodology, validation, writing–review and editing, resources. **Faith B. Friday**: writing–review and editing, resources.

## Ethics Statement

This article does not contain any studies with animals performed by any of the authors. Informed consent was obtained from all authors included in the study.

## Conflicts of Interest

The authors declare no conflicts of interest.

## Peer Review

The peer review history for this article is available at https://publons.com/publon/10.1002/brb3.70820.

## Data Availability

No data were used for the research described in the article.
